# What is “efficiency” in plasma chemical processes?

**DOI:** 10.1016/j.isci.2025.112297

**Published:** 2025-03-29

**Authors:** Charan R. Nallapareddy, Thomas C. Underwood

**Affiliations:** 1 Department of Aerospace Engineering and Engineering Mechanics, The University of Texas at Austin, Austin, TX 78712, USA; 2 Texas Materials Institute, The University of Texas at Austin, Austin, TX 78712, USA

**Keywords:** Chemistry, Chemical engineering

## Abstract

Plasma chemical processes leverage electrical energy for chemical abatement and to rethink how fuels are made, stored, and used throughout the energy economy. These capabilities, along with the flexibility to drive reactions over distributed scales at low temperatures and pressures, offer a pathway to a sustainable circular economy where waste can be converted into value-added resources. However, the scalability and impact of these processes are constrained by the energy costs to drive reactions. Yet there is no standardized framework to evaluate the performance of these processes. Instead, current evaluation methods are ambiguous, inconsistent between sources, and often flawed. In this work, we develop a framework to evaluate the performance of plasma chemical processes that generalizes across all types of chemical reactions. By establishing standardized metrics and benchmarks, this framework aims to accelerate the development and deployment of technologies.

## Highlights

• Developed a standardized framework to assess plasma chemical process performance
• Defined metrics that link reactant conversion and product selectivity to theoretical limits
• Simplified production efficiency to minimize uncertainty from experimental parameters
• Provided benchmarks to assess and compare plasma chemical process performance

## Graphical abstract

**Figure:**
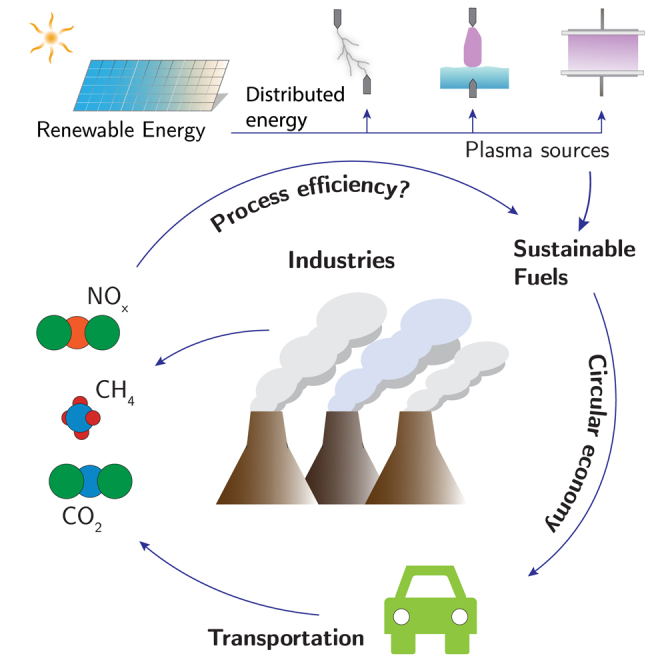

## Introduction

**Figure 1:**
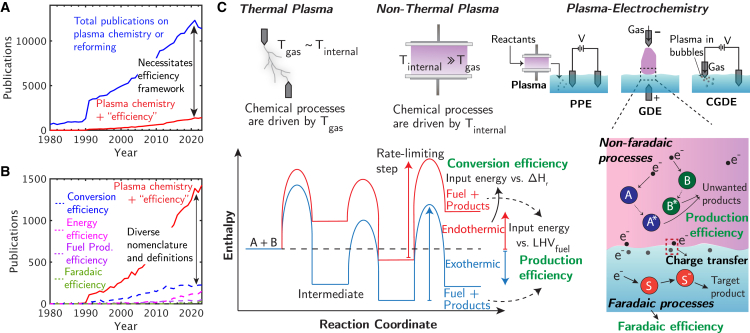
Different efficiency metrics reported in the literature for different types of plasma chemical processes (A) Publications in the plasma chemistry community have increased dramatically over the past ten years, with more than 12,000 publications in 2020 alone (according to the Web of Science platform, Supplemental information Section 4). (B) Yet, most of these publications fail to define efficiency metrics, and of the publications that do define efficiency, different names and definitions limit comparisons among plasma chemical processes. (C) Key performance metrics of a plasma chemical process include conversion efficiency, production efficiency, and faradaic efficiency depending on the type of chemical process. LHV is lower heating value. PPE, GDE, and CGDE refer to post-plasma electrolysis,^4^ glow discharge electrolysis,^5^ and contact glow discharge electrolysis,^5^ respectively.

Plasma chemical processes have the potential to enable a sustainable energy transition where renewable electrical energy (e.g., solar, wind, tidal, etc.) is used to transform greenhouse gases (e.g., CH_4_, CO_2_, NO_x_, etc.) and harmful chemicals (e.g., N_2_O, etc.) into value-added products and fuels.^1^ This embodies a circular economy where waste is converted into usable resources through sustainable processes. In comparison, traditional synthetic pathways, including industrial catalytic processes, require high temperatures (i.e., 1,000 K for steam methane reforming [SRM]), high pressures (i.e., > 30 bars for SMR),^2^ large capital investments, and utilize hydrocarbon process heating that emit sources of carbon into the atmosphere at levels that limit the potential of a circular economy (i.e., 12 kg_CO2,eq_/kg_H2_ for SMR in 2021).^3^ Plasma chemical processes overcome these challenges by using renewable electrical energy to create localized thermodynamic environments (i.e., without bulk scale heating and pressurization) where high temperatures can be formed or reactant molecules can be excited to make reaction pathways favorable and increase the rate of reactions. Additionally, endergonic (ΔG > 0) abatement reactions, such as CO_2_ splitting, are only feasible with highly reactive systems like plasma chemical systems. These systems provide the necessary energy to overcome the thermodynamic barriers of such reactions, enabling processes that would not be possible with finite reaction rates under feasible thermal conditions otherwise. However, the widespread adoption of plasma technologies requires a standardized set of efficiency metrics to evaluate their performance and impact (Figure 1).

**Figure 2:**
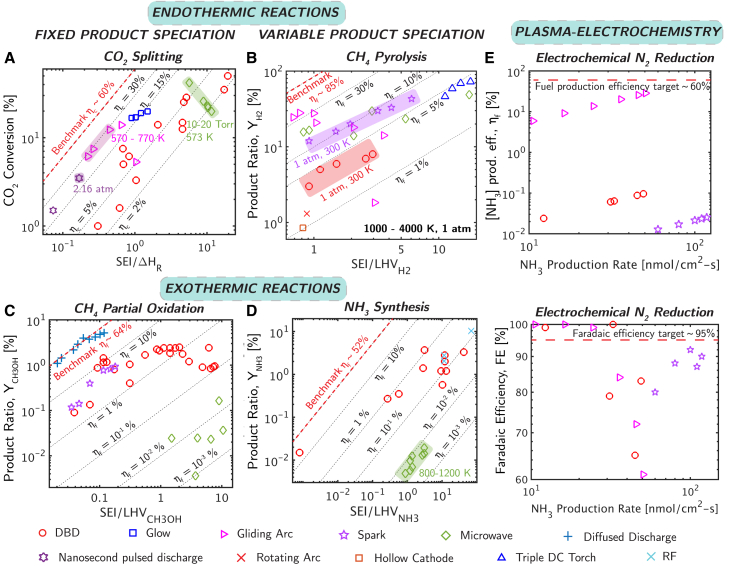
A comparison among various types of plasmas for a diverse range of plasma chemical processes including endothermic, exothermic, and plasma-electrochemical reactions The endothermic reactions include both (A) fixed product composition reactions like CO_2_ splitting^6^^,^^7^^,^^8^^,^^9^^,^^10^^,^^11^^,^^12^^,^^13^^,^^14^^,^^15^^,^^16^^,^^17^ with a benchmark conversion efficiency (η_c_) of 60%,^18^ and (B) variable product composition reactions like CH_4_ pyrolysis^19^^,^^20^^,^^21^^,^^22^ with a benchmark η_f_ ∼85% based on steam reforming.^23^ The exothermic reactions include non-thermal plasma driven processes like (C) CH_4_ partial oxidation^24^^,^^25^^,^^26^^,^^27^^,^^28^^,^^29^^,^^30^^,^^31^^,^^32^^,^^33^^,^^34^ with a benchmark production efficiency (η_f_) of 64% based on steam reforming of methane + Fischer Tropsch synthesis,^34^^,^^35^^,^^36^ and (D) ammonia synthesis^37^^,^^38^^,^^39^^,^^40^^,^^41^^,^^42^^,^^43^^,^^44^^,^^45^^,^^46^^,^^47^^,^^48^ with a benchmark η_f_ of 52% based on Haber-Bosch process.^49^ The plasma-electrochemical reduction of N_2_^4^^,^^50^^,^^51^ in post-plasma electrolysis mode (refer to A) requires both (E) η_f_ for gas-phase plasma treatment (benchmark η_f_ ∼60%)^51^^,^^52^ and faradaic efficiency for electrolysis with a benchmark faradaic efficiency of 95%.^51^^,^^52^ Benchmark metrics evaluation is shown in Supplemental information Section 3. All the data points correspond to near ambient conditions unless indicated. DBD, RF, and ns refer to dielectric barrier, radiofrequency, and nanosecond pulsed discharges, respectively. Refer to Supplemental information Section 1 for definitions for conversion_reactant_, Y, and SEI for different reactor configurations.

**Figure 3:**
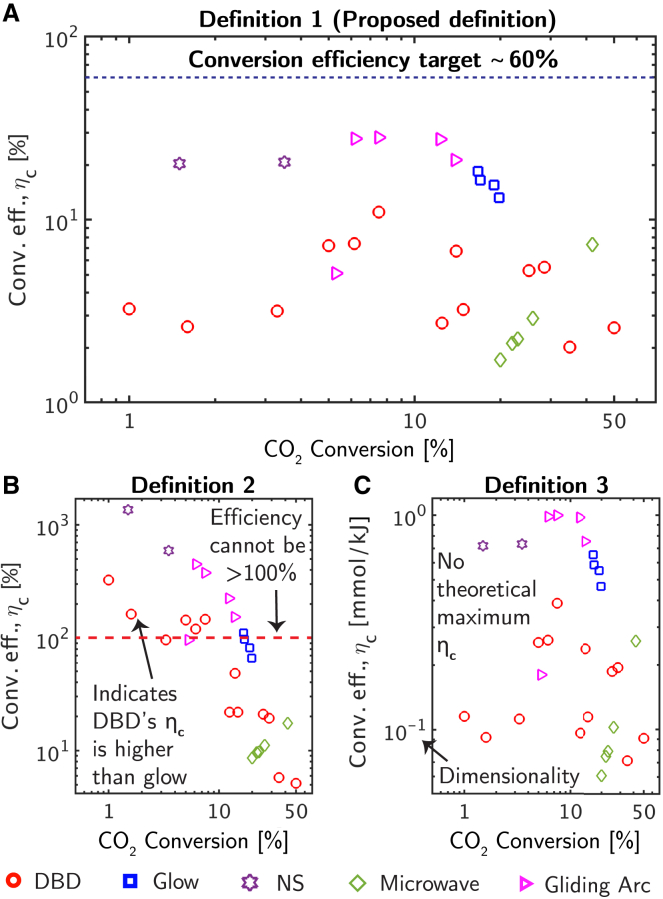
A comparison among various definitions of conversion efficiency for CO_2_ splitting^6^^,^^7^^,^^8^^,^^9^^,^^10^^,^^11^^,^^12^^,^^13^^,^^14^^,^^15^^,^^16^^,^^17^ (A) Figure showing how plasma processes cluster and compare to a benchmark efficiency value of 60% (Supplemental information Section 3) for definition 1 (the proposed definition). (B and C) Inflated (i.e., >100%) conversion efficiency values and different characteristic efficiency patterns with (B) definition 2 and (C) definition 3 with dimensions and no theoretical maximum for η_c_. The data points correspond to various types of non-thermal plasma (e.g., dielectric barrier discharges (DBDs), nanosecond pulsed discharge (NS), and radio frequency (RF)). The definitions 1, 2, and 3 are shown in Equations 1, 2, and 3, respectively.

**Figure 4:**
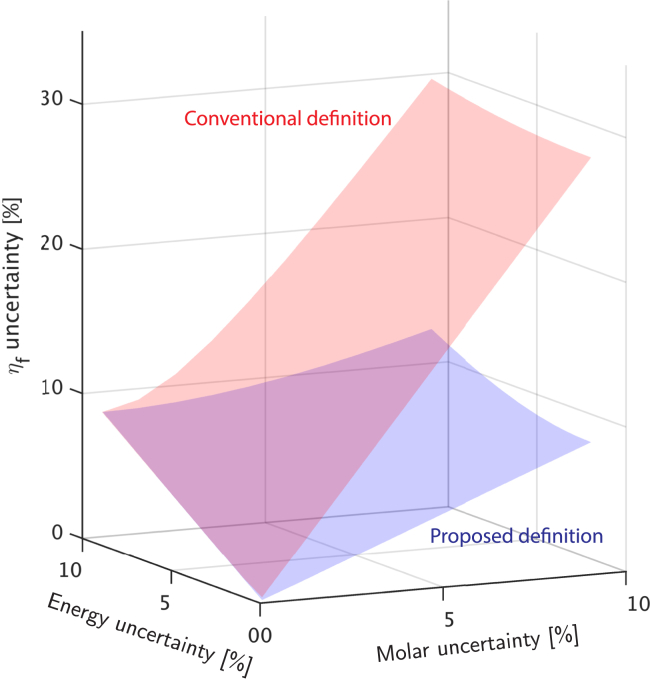
The conventional definition of production efficiency involves 4 interdependent parameters (e.g., quantity of the products generated, SEI, methane conversion and methane composition, Table 1) that must be measured for a given plasma process Each of these parameters contribute uncertainty in quantifying the production efficiency of a process. Instead, we propose a definition with 2 parameters (e.g., product yield and SEI, Equation 4) to reduce this uncertainty for one target product. The evaluation of η_f_ in this plot assumes that O(η_f,proposed_) ∼O(η_f,conventional_) (Figure 5) and a target η_f,proposed_ ∼79% (Figure 5A, Methods Section).

**Figure 5:**
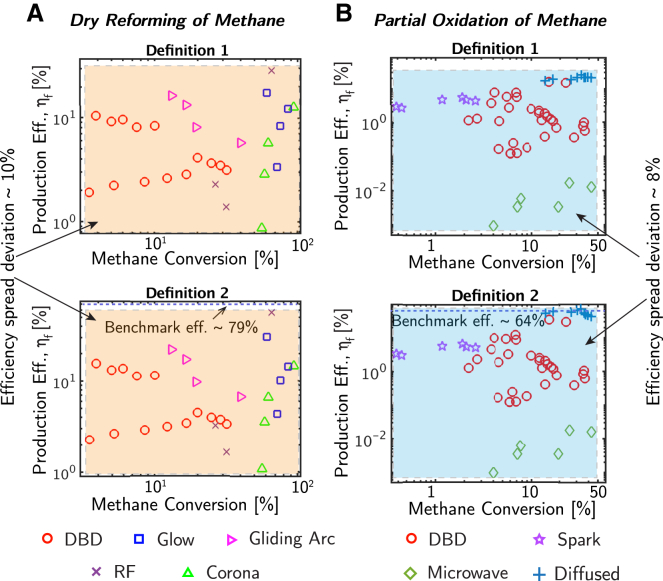
A comparison between the definitions for production efficiency with 4 parameters and 2 parameters (Table 1, Methods section) (A) Endothermic non-thermal plasma reaction (dry reforming of methane to syngas, and the target product here refers to CO),^54^^,^^55^^,^^59^^,^^60^^,^^61^^,^^62^^,^^63^^,^^64^^,^^65^^,^^66^^,^^67^^,^^68^ with a benchmark efficiency of 79%.^69^ (B) Exothermic non-thermal plasma reaction (partial oxidation of methane to methanol)^24^^,^^25^^,^^26^^,^^27^^,^^28^^,^^29^^,^^30^^,^^31^^,^^32^^,^^33^^,^^34^ with a benchmark efficiency of 64% (Supplemental information Section 3).^34^^,^^35^^,^^36^ The proposed definition (Equation 4) shows less than a 10% standard deviation in efficiency compared to the more conventional definition (i.e., $\eta_{f}$ from Table 1) with a greater number of parameters. Here, definition 1 and 2 refer to $\eta_{f}$ definitions from Table 1 with and without the conversion term in the denominator, respectively.

With this potential, research into plasma chemistry continues to expand to target industrial applications, with more than 12,000 papers published in 2020 alone (according to the Web of Science database, Figure 1A, Figure 1, Figure 2, Figure 3, Figure 4, Figure 5 Section 4). Yet efficiency is mentioned in less than 15% of these papers and, of those that do, only 30% report a number. Instead, existing studies reference a range of performance metrics that are not standardized across different types of reactions and plasma chemical systems (Figure 1B). For example, the definition of “product selectivity”—a widely used performance indicator—can be ambiguous depending on which atoms are present, reactants are monitored, and products are measured in a reaction (Supplemental information Section 1). While this metric provides valuable information regarding a plasma chemical process, it can lead to inconsistent interpretations of processes when different atoms are used as reference points. For instance, calculating selectivity based on carbon or oxygen in a hydrocarbon oxidation reaction can yield different values and hinder the comparison and optimization of technologies. This illustrates the need for a standardized framework to evaluate the efficiency of plasma chemical processes, including the identification of key metrics that are tailored for each type of chemical reaction.

In this work, we propose a standardized framework for evaluating plasma chemical processes across diverse thermodynamic conditions. We aim to describe a set of criteria to evaluate plasma chemical processes and assess their scalability toward industrial conditions. The proposed framework focuses on plasma processes exclusively that aim at minimizing energy costs of any plasma chemical process. We believe that a performance metric should not only report the obtained quantities (like reactant conversion and product yield) for a given energy but also compare them to their theoretically possible limits. Therefore, the framework offers three universal, dimensionless metrics, each with a maximum value of 100% by comparing the quantities to their theoretical maximums: conversion efficiency ($\eta_{c}$), production efficiency (sometimes referred to as fuel production efficiency) ($\eta_{f}$), and faradaic efficiency (FE) (Table 1). These are thermodynamic metrics and do not account for capital costs or reactor-specific factors, such as reactor volume, flow rates, or kinetic aspects (i.e., reaction rates). These metrics are then applied to analyze key plasma chemical processes, including endothermic, exothermic, and plasma-electrochemical reactions (Figure 2) and help identify how different plasma processes cluster based on their thermodynamics and excitation environment (Figures 2, 5). We show how usage of different definitions can lead to misinterpretation of the performance. We discuss how performance metrics with large number of parameters are prone to high uncertainty propagation, and we simplify those metrics for easier adaptability and to reduce the uncertainty during evaluation. Finally, we apply our framework to processes of interest, including CO_2_ splitting, dry reforming of methane, ammonia synthesis, methane pyrolysis, and partial oxidation of methane, to evaluate performance targets for industrial adoption (Supplemental information Section 3; Figure 2), rank, and classify plasma chemical technologies.

**Table 1:** Proposed efficiency framework to evaluate the performance of plasma chemical processes

Efficiency metric	Efficiency definition	Type I	Type II	Type III	Type IV
Conversion efficiency ($\eta_{c}$)	$\eta_{c}=\frac{\Delta {\hat{H}}_{r}\sum_{i=1}^{\text{reactants}}\alpha_{i}{\;\text{conversion}}_{i}}{\text{SEI}}$	Yes^#^			
Production efficiency ($\eta_{f}$)	$\eta_{f}=\left\{ \begin{array}{c} \frac{{\text{LHV}}_{\text{target}\;\text{product}}Y_{\text{target}\;\text{product}}\;}{\text{SEI}}\;\text{if}\;N_{\text{target productl}\;}=1^{⊥}\\ \frac{\sum_{i=1}^{\text{products}}{\text{LHV}}_{i}Y_{i}}{\text{SEI}+\sum_{i=1}^{\text{reactants}}{\text{conversion}}_{i}\alpha_{i}{\text{LHV}}_{i}}\;\text{if}\;N_{\text{target product}\;}\geq 1\;\left(\text{or}\right)\;\\ \text{when}\;\text{accurate}\;\text{estimation}\;\text{of}\;\eta_{f}\;\text{is}\;\text{needed}. \end{array}\right.$				
			Yes	Yes	Yes
Faradaic efficiency (FE)	$\text{FE}=\frac{z\;{\hat{n}}_{\text{product},\text{faradaic}}\;F}{\int I\;\text{dt}}$				Yes^∗^

$\Delta {\hat{H}}_{r}$: Reaction enthalpy; $\alpha_{i}$: mole fraction of species i; conversion: reactant conversion (Supplemental information Section 1); SEI: Specific energy input (energy per unit reactant molecule, Supplemental information Section 1); N_target product_: Number of target products; Y: product ratio (the amount of target product produced relative to the initial reactants, Supplemental information Section 1); LHV_target__product_: Lower heating value; z: Number of electrons required for a mole of product; ${\hat{n}}_{\text{product},\text{faradaic}}$: Faradaic yield or number of moles of target product produced via faradaic processes; $\bar{\text{power}}$: Average power; F: Faraday constant; I: Current and ∫Idt: Total charge passed. Type I, II, III and IV indicate endothermic with fixed product distribution or known $\Delta {\hat{H}}_{r}$, endothermic with variable production distribution or unknown $\Delta {\hat{H}}_{r}$, exothermic and plasma-electrochemical reactions respectively. ^∗^Symbol indicates applicability to only faradaic processes and faradaic product yield. ^#^Symbol indicates the equivalence between conversion and production efficiencies for Type I reactions when the reactants have no LHVs (Supplemental information Section 6), reducing redundancy in reporting multiple efficiency metrics. ^⊥^Symbol indicates that the definition is useful even in the case of two stoichiometrically constrained products like CO + H_2_ in SMR (Supplemental information Section 3). In a flow reactor, Y_i_ = $\frac{{\hat{\dot{n}}}_{\text{target}\;\text{product}}}{\sum_{i=1}^{\text{reactants}}{\hat{\dot{n}}}_{i,\mathrm{initial}}}$; conversion_i__=_$\frac{{\hat{\dot{n}}}_{\text{reactant},\text{initial}}\;-\;{\hat{\dot{n}}}_{\text{reactant},\text{final}}}{{\hat{\dot{n}}}_{\text{reactant},\text{initial}}}$; $\text{SEI}=\frac{\bar{\text{power}}}{\sum_{i=1}^{\text{reactants}}{\hat{\dot{n}}}_{i,\text{initial}}}$; $\alpha_{i}=\frac{{\hat{\dot{n}}}_{i,\text{initial}\;\text{reactant}}}{\sum_{i=1}^{\text{reactants}}{\hat{\dot{n}}}_{i,\text{initial}}}$, where $\hat{\dot{n}}$ = molar flow rate. Corresponding parameters for a batch mode reactor and more details are provided in Supplemental information Section 1.

### The problem: Useful but incomplete performance metrics

The performance of plasma chemical processes depends on several factors that include the type of reaction, conversion of reactants (i.e., the destruction and removal efficiency, DRE, in the case of abatement processes), yield of products, rate kinetics, and process input costs (e.g., electrical energy, etc.). While plasmas can lower activation barriers and accelerate chemical reactions, the scalability of these processes are constrained by the energy costs that are required to drive them, the amount of desirable products that can be produced, and the selectivity by which they can be generated. Given these constraints, processes are evaluated typically by reporting the energy input to sustain a plasma, reactant conversion, and product selectivity (or yield) for a given reaction. Relying only on these metrics does not show the maximum product yield (or selectivity) or reactant conversion that is possible for a given energy input. In other words, these parameters do not inform how much reactant could be converted or how much product could be produced for a given amount of energy. We believe that performance metrics should evaluate these quantities relative to their theoretical energy conservation limits (i.e., the maximum quantity that can be produced assuming no energy losses) for a given energy input.

There are additional limitations that impact the applicability of these metrics in assessing plasma chemical processes. One example is the use of product selectivity, which is defined as the ratio of the moles of a desired product to the total moles of all products produced in a reaction. While this definition is general, its practical implementation in plasma systems is challenging. Unlike thermo-catalytic reactions, which typically produce fewer byproducts due to their more controlled reaction pathways, plasma-driven reactions often generate a large pool of reactive species, leading to branching reactions and multiple major products that must be measured individually to quantify selectivity accurately. Additionally, when a reaction involves mole-producing or consuming pathways, the conservation of moles between reactants and products no longer holds. Consequently, the amount of product molecules cannot be determined directly from the number of moles of converted reactants.

Other forms of product selectivity have been adopted in the literature to reduce the complexity of measuring it.^53^ These include methods to track the exchange of atoms between reactants and products.^54^^,^^55^ While there is nothing fundamentally wrong with this definition, we want to highlight a critical limitation: the lack of standardization across studies when reporting selectivity. To illustrate how this is a problem, consider a plasma-driven methane partial oxidation reaction with an unknown product distribution,

$$CH_{4}+O_{2}\;\to \;a\;CH_{3}\text{OH}+b\;H_{2}+c\;\text{CO}+d\;CO_{2}+{\;e\;C}_{2}H_{6}+\text{other}\;\text{products}$$

Now, consider if a plasma produces equal moles of ethane and methanol, ${\hat{\dot{n}}}_{C_{2}H_{6}}={\hat{\dot{n}}}_{CH_{3}\text{OH}}\text{.}$ This should mean the selectivity of ethane (C_2_H_6_, $S_{C_{2}H_{6}}$) is equal to methanol (CH_3_OH, $S_{CH_{3}\text{OH}}$),

$$S_{C_{2}H_{6}}=\frac{{\hat{\dot{n}}}_{C_{2}H_{6}}}{\sum_{i=1}^{\text{products}}{\hat{\dot{n}}}_{i}}=\frac{{\hat{\dot{n}}}_{CH_{3}\text{OH}}}{\sum_{i=1}^{\text{products}}{\hat{\dot{n}}}_{i}}=S_{CH_{3}\text{OH}}\text{.}$$

However, a product selectivity based on the moles of atomic carbon converted ($\Delta {\hat{\dot{n}}}_{CH_{4}})$ results in a higher selectivity value for C_2_H_6_, $S_{C_{2}H_{6}}^{C}=\frac{2{\hat{\dot{n}}}_{C_{2}H_{6}}}{\Delta {\hat{\dot{n}}}_{CH_{4}}}$, than CH_3_OH, $S_{CH_{3}\text{OH}}^{C}=\frac{{\hat{\dot{n}}}_{CH_{3}\text{OH}}}{\Delta {\hat{\dot{n}}}_{CH_{4}}}\text{,}$ where ${\hat{\dot{n}}}_{CH_{3}\text{OH}}$ and $2{\hat{\dot{n}}}_{C_{2}H_{6}}$ represent the moles of carbon in the ethane (${\hat{\dot{n}}}_{C_{2}H_{6}}$) and methanol (${\hat{\dot{n}}}_{CH_{3}\text{OH}}$) formed respectively. Similarly, a product selectivity based on hydrogen atom as the reference shows different values: $S_{C_{2}H_{6}}^{H}=\frac{6{\hat{\dot{n}}}_{C_{2}H_{6}}}{4\Delta {\hat{\dot{n}}}_{CH_{4}}}$ and $S_{CH_{3}\text{OH}}^{H}=\frac{{\hat{\dot{n}}}_{CH_{3}\text{OH}}}{\Delta {\hat{\dot{n}}}_{CH_{4}}}\text{.}$ This conclusion follows from the definition of the selectivity metric (Supplemental information Section 1) and illustrates how inconsistent definitions can result in different interpretations of performance in plasma processes, especially when vaguely referred to as “selectivity”. While atom-based selectivity provides insight into atom distribution within a system, and rate-based selectivity can, in some cases, offer more meaningful insights than molar selectivity, the “correct” definition ultimately depends on the specific problem at hand. Given this lack of standardization across studies when reporting selectivity, a framework is needed to unify how plasma chemical processes are evaluated, including how performance is ranked, and energy costs are included.

### Proposed efficiency framework

In this generalized framework (Table 1), each metric combines performance descriptors (e.g., conversion [or DRE], yield, energy costs, etc.) into a set of universal efficiency metrics that apply to different classes of plasma chemical reactions. The framework focuses only on the performance of the plasma and does not integrate system level considerations like compression stages, heat integration, and the recovery of excess heat. Each metric in the framework is bounded (i.e., <100%) and non-dimensional by defining it as the ratio of a measured property (e.g., reactant conversion, product yield, etc.) to its theoretical limit. For example, conversion efficiency and production efficiency quantify how energy is being spent to convert reactants and produce desirable products in plasmas. These metrics encompass the reactant conversion (the ratio of a converted reactant to its initial amount), the product ratio (Y, the amount of a target product produced relative to the initial reactants), specific energy input (SEI, energy input per unit reactant molecule), reactant composition (α, the ratio of a specific reactant to the total initial reactants), reaction enthalpy ($\Delta {\hat{H}}_{r}$), and the lower heating value (LHV). Energy input to the plasma is calculated by integrating the input electrical power over the runtime. This calculation reflects the total energy delivered to the plasma that can be used to (1) drive chemical reactions (i.e., stored in chemical bonds), (2) heat the gas through relaxation processes (i.e., sensible heating), (3) emit radiation, and (4) induce other transport induced energetic processes. FE quantifies how electrons are being used to form desirable products in faradaic processes. While each metric is distinct, we generalize them into a framework by describing which type of plasma chemical reaction each efficiency applies to. This includes four separate reaction categories: (type I) endothermic reactions with a well-defined product distribution, selectivity, and a known heat of reaction (e.g., CO_2_ splitting [Figure 2A]), (type II) endothermic reactions with an unknown product distribution (e.g., CH_4_ pyrolysis [Figure 2B], CH_4_ dry reforming), (type III) exothermic reactions (e.g., CH_4_ partial oxidation [Figure 2C], NH_3_ synthesis [Figure 2D]), and (type IV) plasma-electrochemical reactions (e.g., electrochemical reduction of N_2_ [Figures 2E and 2F]). Benchmark efficiencies for plasma chemical processes in each reaction type are evaluated by analyzing costs of existing approaches (e.g., SMR and Haber process) and competing solar-driven technologies (Supplemental information Section 3, Table S2; Figure 2).

The use of efficiency metrics to characterize a plasma requires more consideration than just measuring the state and energy cost of a chemical process. The correct efficiency metric must be selected for a chemical reaction of interest. Take conversion efficiency as an example, a metric that quantifies the conversion of reactants relative to their theoretical limit for a given amount of energy input (i.e., SEI/ $\Delta {\hat{H}}_{r}$). Evaluating conversion efficiency requires quantifying the theoretical limit of conversion, something that is useful for reactions that are both endothermic and have a known product distribution (type I, Table 1). In particular, only type I endothermic reactions have a defined $\Delta {\hat{H}}_{r}$. Select abatement reactions, like CO_2_ splitting, follow these requirements (i.e., fixed CO and O_2_ selectivity^6^ leading to a fixed $\Delta {\hat{H}}_{r}$) and enable conversion efficiency to be measured and remain bounded (i.e., <100%). The same is not true for endothermic reactions involving an unknown and varying distribution of products (type II, Table 1). CH_4_ pyrolysis is one such reaction with a product distribution (e.g., soot, H_2_, C_2_H_6_, C_2_H_2_ etc.) that changes depending on the type of plasma and the thermodynamic environment it generates. This means that $\Delta {\hat{H}}_{r}$ for the reaction is unknown and conversion efficiency cannot be bounded.

Production efficiency is a more general metric that evaluates how energy is used within a plasma to form products of interest. Unlike conversion efficiency, production efficiency only considers the amount of target fuels/products (or products with a non-zero LHV for abatement processes, Supplemental information Section 8) that are produced in a reaction for a given energy input. This simplifies the application of production efficiency to a broader class of chemical reactions as only information about product fuels is required and not the entire distribution of product species. To compute efficiency, the metric compares the yield of target fuels/products with the theoretical limit for their yield assuming that all the plasma energy is stored in the products and sensible losses are ignored. This theoretical limit is determined by the ratio of energy input to the LHV of the target fuels or products. The LHV represents the usable (or combustible) energy stored in a fuel, and under ideal conditions with no losses, the total input energy should be fully converted into this usable energy. Thus, this ratio establishes the theoretical maximum yield achievable for a given energy input.

Some types of reactions should report multiple types of efficiencies (Table 1) while others should only report a single metric. For example, in type I endothermic reactions, like CO_2_ splitting, both conversion efficiency and production efficiency are identical and either can be reported (Supplemental information Section 6). However, only production efficiency is valid for endothermic reactions with an unknown product distribution (type II). A survey of current literature shows this distinction is not clear in the plasma community, in fact more than 45% of publications report conversion efficiency instead of production efficiency for type II reactions (Figure S1, Supplemental information).

Plasma electrochemical processes are the final class of reactions considered in the proposed efficiency framework (type IV). These processes can operate in various configurations including glow discharge electrolysis (GDE),^5^^,^^56^ contact glow discharge electrolysis (CGDE),^5^ and post-plasma electrolysis (PPE)^4^ (Figure 1C). Each configuration differs by where electrodes are placed (i.e., one submerged or both submerged) or when plasma is exposed to an electrolyte (i.e., before or after performing electrolysis). Performance in these systems is evaluated conventionally using FE, a measure of how efficient electrons are used to form products in faradaic reactions.^57^ Although predominant, this metric is incomplete in evaluating the performance of plasma electrochemical processes and requires careful consideration to use correctly in plasma systems. For example, in a PPE configuration where plasma treatment and electrolysis are separate steps, production efficiency and FE should be reported together to evaluate the performance of a process (Figures 2D and 2E). In other configurations, including GDE and CGDE schemes, a plasma-liquid interface is introduced that makes separating plasma-driven and electrochemical-driven reactions difficult as both faradaic and non-faradaic processes happen simultaneously. This can result in FEs being greater than 100%^56^ when target products that are produced from non-faradaic pathways are included which can make assigning a target/benchmark efficiency challenging. To get around this issue, researchers should report production efficiency, and when evaluating FE, separate product yields from faradaic and non-faradaic processes.^5^ In the cases where that is not possible, reporting production efficiency alone can be sufficient.

The efficiency framework enables the classification and ranking of performance for various plasma chemical processes that come under type 1–IV. This includes identifying how plasma technologies cluster and establishing benchmark values for each metric (Figure 2). For example, in both CO_2_ splitting and dry reforming of methane, DBD displayed the lowest conversion efficiency compared to other forms of non-thermal plasmas. For CO_2_ splitting, arc discharge, with its gas temperature range (570–770 K) promoting both thermal and non-thermal CO_2_ dissociation (via ladder climbing mechanism), has the potential to reach the target conversion efficiency of 60%.

### Standardizing the definitions of efficiency metrics

Standardization among efficiency metrics allows plasma chemical processes to be evaluated using a common set of criteria. Without standardization, differences among definitions and how they are applied can lead to inconsistencies in how processes are compared, innovations are evaluated, and resources are allocated, especially for scaling up to industrial conditions. To illustrate this issue, we surveyed literature to identify different names and definitions that are used to describe conversion efficiency. Across these papers, we found more than nine different definitions and four different names (Table S1, Supplemental information), many of which were dimensional or were not bounded to be <100%.

Different definitions among efficiency metrics can change how the performance of plasma chemical processes clusters for a given chemical reaction. As one example, consider the splitting of CO_2_, a type I reaction (Table 1), a condition where conversion efficiency and production efficiency are equal (Supplemental information Section 6). However, the evaluation of conversion efficiency varies among sources, with three common definitions including,

(Equation 1) $$\text{Definition}\;1:\eta_{c}=\frac{\Delta {\hat{H}}_{r}\sum_{i}^{\text{reactants}}\alpha_{i}\;\text{conversio}n_{i}}{\text{SEI}},$$

(Equation 2) $$\text{Definition}\;2:\eta_{c}=\frac{\Delta {\hat{H}}_{r}}{\text{SEI}},$$

(Equation 3) $$\text{Definition}\;3:\eta_{c}=\frac{\sum_{i}^{\text{reactants}}\alpha_{i}\;\text{conversio}n_{i}}{\text{SEI}}.$$

These definitions change how plasma-driven CO_2_ splitting is evaluated. Depending on which definition is used, the performance of different plasma sources (e.g., non-thermal, thermal, etc.) varies (Figure 3). Definition 1, which is the proposed definition for conversion efficiency in our framework, is both non-dimensional and thermodynamically bounded to 100%. This is not the case with definitions 2 and 3 (Equations 2 and 3) nor is it with other definitions in the literature (Table S1, Supplemental information). Not only are these definitions unbounded (in the cases of both definition 2 and 3) and dimensional (in the case of definition 3), which makes assigning target/benchmark metrics challenging, but they also predict different plasma sources have better performance (Figure 3B). This can result in misleading comparisons in the literature where two different definitions for efficiency are used.

### Simplifying the evaluation of efficiency metrics

Definitions for efficiency metrics should be as easy to use and straightforward as possible, remaining bounded and dimensionless, to encourage their widespread adoption in the literature. Metrics that depend on many parameters are difficult to evaluate, require more measurements, and therefore lead to higher uncertainty in their evaluation. For instance, conventional definitions of production efficiency with more than one target fuel rely on multiple parameters, including Y, SEI, conversion, LHV, and α (Table 1). It has been shown that evaluating reactant conversion accurately in a flow reactor involves the measurement of even more experimental parameters.^53^ These dependencies introduce measurement uncertainty, which can skew results and obscure the process performance (Figure 4). Without considering these uncertainties, which can exceed 5–10% in gas chromatography injection systems,^58^ inaccurate estimates of production efficiency can be calculated and outliers in process performance can be reported in the literature.

To overcome this challenge, we outline a simpler definition for production efficiency that features a smaller number of parameters. This definition is applicable when there is a single product of interest (N_target product_ = 1), one combustible reactant (e.g., CH_4_, H_2_, etc.), and/or an oxidizing reactant (e.g., CO_2_, N_2_, O_2_, etc.), a case that happens in many plasma chemical processes where high selectivity toward a single value-added product is desired (e.g., NH_3_ synthesis, CH_4_ partial oxidation to methanol, etc.). This definition remains useful even in multi-reactant plasma-driven endothermic processes, like steam reforming and dry reforming of methane that target two products/fuels (i.e., syngas, CO + H_2_), where the selective production of multiple fuels improves simultaneously due to stoichiometric constraints (Supplemental information Section 3). Since conversion is a function of SEI, the definition of $\eta_{f}$ can be simplified to a definition with fewer (i.e., two) independent parameters (Supplemental information Section 5),

(Equation 4) $$\begin{array}{c} \eta_{f,\text{proposed}\;}=\frac{\text{LH}V_{\text{product}}Y_{\text{product}}}{\text{SEI}}\text{.} \end{array}$$

This definition (Equation 4) features key advantages compared to the more general expression for production efficiency (Table 1) with $N_{\text{target}\;\text{product}}>1$. These include: (1) the definition reduces the number of parameters that must be measured experimentally while maintaining the same clustering (with <10% standard deviation in the spread) among plasma chemical approaches (Figure 5). (2) The definition decreases the uncertainty in estimating production efficiency by lowering the number of measured parameters (Figure 4). Together, these features enable an easier to implement and reliable expression for calculating production efficiency, especially in conditions that have limited bandwidth to measure process descriptors (i.e., conversion, yield, and energy costs). Our analysis shows that the simplified definition for production efficiency (Equation 4) provides the same clustering of efficiency values as the conventional definition across various plasma types and reaction categories (endothermic and exothermic) (Figure 5).

While all the definitions in the framework are thermodynamic metrics, to ensure boundedness in the proposed definition for production efficiency, one key condition must be satisfied: it is the relation between SEI and reactant conversion. In exothermic reactions, conversion can occur thermodynamically without any energy input over infinite time. However, in plasma chemical processes with finite reaction rates, energy input from the plasma (SEI) is required to overcome energy barriers for the reaction to proceed. SEI drives conversion, enabling the use of a simplified definition to evaluate production efficiency (Supplemental information Section 5). This means that SEI cannot be zero or decoupled from conversion values in a plasma, ensuring the definition remains meaningful and bounded. Additionally, to ensure equivalence with the original definition, the condition SEI > α Conversion_reactant_ LHV_reactant_ must hold. In industrial plasma catalytic systems, this is naturally satisfied: at low conversions, conversion increases linearly with SEI, allowing the denominator of the original definition to simplify to SEI alone; at high conversions, conversion plateaus with SEI, maintaining the validity of the condition (Figure 5 and Supplemental information Section 5). This behavior, combined with inevitable energy losses and the intrinsic relationship between SEI and conversion, guarantees the boundedness, reliability, and broad applicability of the proposed framework.

### Conclusion

In this work, we establish a framework to evaluate the performance of plasma chemical processes as a method to synthesize sustainable fuels and abate harmful chemicals (Table 1). With this framework, we identify efficiency metrics and describe a set of criteria (i.e., metrics must be dimensionless and have a maximum theoretical value of 100%) to eliminate diverse, inconsistent, and inaccurate nomenclature in the literature. The goal of this framework is to standardize the evaluation of plasma chemical processes, including endothermic, exothermic, and plasma-electrochemical reactions. In particular, we describe efficiency metrics that quantify reactant conversion, product yield, and charge consumption compared to their theoretical limits respectively. While these metrics are valuable for assessing plasma chemical processes, they are not the only available evaluation metrics. Product selectivity based on mass or rate can also serve as a useful metric. However, the lack of standardization in these definitions presents a challenge, which we addressed by introducing the proposed framework.

Each metric is described in a form that limits its complexity while improving the accuracy over which it can be measured. We apply this framework to a diverse range of endothermic (e.g., CH_4_ pyrolysis and CO_2_ splitting), exothermic (e.g., CH_4_ partial oxidation and NH_3_ production), and plasma electrochemical reactions (e.g., NH_3_/NH_4_^+^ production) to highlight which metrics are critical, what each metric implies, how they should be used, and how plasma environments cluster based on their thermodynamics and excitation environment (Figures 2 and 5). Together, the advancements in our framework show a pathway to categorize plasma technologies, evaluate process advancements, and identify performance criteria for different plasma technologies.

### Limitations of the study

The framework in this paper focuses on assessing the efficiency of plasma chemical systems, where reactions are driven by the addition of energy. The simplified production efficiency definition (Equation 4) is valid only when there is a single product of interest, one combustible reactant (e.g., CH_4_ and H_2_), and/or an oxidizing reactant (e.g., CO_2_, N_2_, and O_2_), conditions commonly found in plasma chemical processes. Additionally, this definition holds only when SEI ≫ α × Conversion_reactant_ × LHV_reactant_, ensuring efficiency does not exceed 100%. Otherwise, the more general expression for production efficiency must be used (Table 1). For instance, at an NH_3_ production rate of 1.6 × 10^−5^ mol/min, corresponding to a 2.4% N_2_ conversion at 136 W of applied power, with an H_2_/N_2_ molar ratio of 3 and a total flow rate of 30 cm^3^/min, the SEI is approximately 6,100 kJ/mol. Meanwhile, the term α × Conversion_reactant_ × LHV_reactant_ evaluates to ∼13 kJ/mol. As a result, the conventional definition of efficiency yields 3.73%, while the simplified definition gives 3.74%.

## Resource availability

### Lead contact

Thomas C. Underwood, Department of Aerospace Engineering, The University of Texas at Austin, Austin, Texas 78712, United States; Texas Materials Institute, Email: (thomas.underwood@utexas.edu).

### Materials availability

No new experimental data were generated in this study. All relevant resources have been provided in the main manuscript or the supplementary materials. Further information and requests for resources should be directed to and will be fulfilled by the lead contact.

### Data and code availability

All relevant data supporting the findings of this manuscript are included in the manuscript or supplemental information. The paper does not report original code. Any additional information required to reanalyze the data reported in this paper is available from the lead contact upon reasonable request.

## Acknowledgments

We acknowledge support from the U.S. Department of Energy (DOE) under grant number DE-SC0024437, with Dr. Nirmal Podder as the program manager.

## Author contributions

C.R.N. and T.C.U. conceptualized the study, conducted the analysis, and wrote the manuscript.

## Declaration of interests

The authors declare no competing interests.

## STAR★Methods

### Key resources table

**Table:** 

REAGENT or RESOURCE	SOURCE	IDENTIFIER
**Software and algorithms**		

MATLAB R2024b	Mathworks	https://www.mathworks.com/products/matlab.html

**Other**		

Web of Science	Clarivate Analytics	https://www.webofscience.com/wos/woscc/basic-search

### Method details

#### Benchmark values for efficiency metrics

The efficiency framework enables the classification and ranking of performance for any plasma chemical process, including identifying how technologies cluster and establishing benchmark values for each metric (Figure 2). In particular, benchmark values establish performance thresholds for plasma technologies to be competitive with existing industrial processes. These values can be estimated using a combination of techno-economic analyses (TEA) from the literature and comparisons with the performance and economics of established processes. For example, in CO_2_ splitting, a benchmark conversion efficiency of 60% is needed for plasma processes to be competitive with solar-driven technologies that achieve 20% solar-to-fuel conversion efficiency.^70^ In CH4 pyrolysis, where H2 is the desired product, steam methane reforming (SMR) serves as a benchmark. With a total energy consumption of 141.48 MJ/kgH2 (including electricity, cooling, and heat exchange) for SMR,^23^ a production efficiency of 85% is needed for plasma-driven CH4 pyrolysis to be competitive over industrial scales. For CH4 partial oxidation to methanol, the two-step steam methane reforming + Fischer Tropsch process serves as the industrial standard for producing methanol (or other liquid oxygenates) from CH4. Energy consumption for unit methanol production, 30.8 kJ/gMeOH,^36^ was used to obtain the production efficiency of 64% as detailed below. In NH3 synthesis, the Haber-Bosch process serves as the industrial standard and benchmark for performance. Haber-Bosch NH3 production typically consumes 9-11 kWh/kgNH3,^44^^,^^49^ corresponding to a benchmark production efficiency of ∼52% for a competitive plasma chemical NH3 synthesis technology. For plasma-catalytic dry reforming of methane, using Gibbs free energy minimizing approach, a benchmark electricity demand of 49.7 kWh/kmolsyngas is established (equivalent to 79% production efficiency) for competitiveness with the thermal catalytic approach.^69^ Finally, for the electrochemical N2 reduction process, which involves both faradaic and non-faradaic yields, benchmark values of 95% faradaic efficiency and 8.64 kWh/kgNH3 energy consumption (equivalent to 60% production efficiency) are set to be competitive with the conventional method. These benchmarks are derived from TEA of direct electrochemical N2 reduction (i.e., conventional electrochemical process).^51^^,^^52^

##### CH_4_ pyrolysis to H_2_

As H_2_ is the target fuel in methane pyrolysis, we considered steam methane reforming (SMR) as a performance standard for the production of H_2_. SMR is the current industrial standard method to produce H_2_ and is a standard that plasma chemical processes must match to be adopted broadly.^71^

Based on the operating parameters of SMR, the energy consumption to produce H_2_ involves (Jose Osorio-Tejada et al.),^23^

$$\text{Electricity}=0.59\frac{\text{kWh}}{{\text{kg}}_{H2}}\text{,}$$

$$\text{Cooling}\;\text{energy}=52.63\;\frac{\text{MJ}}{kg_{H2}}\text{,}$$

$$\text{Heat}=86.36\;\frac{\text{MJ}}{kg_{H2}}\text{,}$$

and the total energy consumption is 141.48 MJ/kg_H2_. This serves as a target for the SEI of plasma technologies.

The following definition was used for evaluating conversion efficiency,

$$\text{Production}\;\text{efficiency}=\frac{\text{moles}\;\text{of}\;\text{fuel}\times {\text{LHV}}_{\text{fuel}}}{\text{total}\;\text{energy}\;\text{input}}\text{.}$$

Here the target fuel is H_2_. Therefore,

$$\text{Production}\;\text{efficiency}=\frac{\text{moles}\;\text{of}\;H_{2}\times {\text{LHV}}_{H_{2}}}{\text{total}\;\text{energy}\;\text{input}}\text{.}$$

Since the total energy consumption per mole of H_2_ production is 141.48 MJ/kg_H2_.

$$\text{Production}\;\text{efficiency}\;\left[\%\right]=\frac{\;240\frac{\text{kJ}}{\text{mol}}}{141.48\frac{\text{kJ}}{g_{H2}}\times 2\frac{g_{H2}}{\text{mol}}}\times 100\;∼\;85\%$$

##### CO_2_ splitting

For CO_2_ splitting, a benchmark conversion efficiency of 60% was chosen from Snoeckx et al.^18^ and Bogaerts et al.^70^ This benchmark metric is based on novel approaches that implement renewable solar energy to convert CO_2_ thermochemically. A 20% solar-to-fuel conversion efficiency is said to be industrially competitive,^70^ and as a result, a conversion efficiency of 60-80% was stated to be necessary by Bogaerts et al.^70^ for plasma-based CO_2_ splitting to be commercial and compete with other technologies that utilize solar energy.

##### CH_4_ partial oxidation to methanol

The benchmark production efficiency for methanol (MeOH) production from methane partial oxidation was based on the steam methane reforming + Fischer Tropsch process. Nallapareddy et al.^34^ evaluates energy consumption in SMR + FT process based on techno-economic analysis shown in Lange et al. and Blumberg et al.^35^^,^^36^

Energy consumption for methanol production = 30.8 kJ/g_MeOH_.

$$\text{Production}\;\text{efficiency}=\frac{\text{moles}\;\text{of}\;\text{fuel}\times {\text{LHV}}_{\text{fuel}}}{\text{total}\;\text{energy}\;\text{input}}\text{.}$$

$$=\frac{\text{LH}V_{\text{MeOH}}}{\text{Energy}\;\text{consumption}\;\text{per}\;\text{mole}\;\text{of}\;\text{MeOH}}\;\text{.}$$

$$\text{Production}\;\text{efficiency}\;\left[\%\right]=\frac{636.8\frac{\text{kJ}}{\text{mol}}}{30.8\frac{\text{kJ}}{g_{\text{MeOH}}}\times 32\frac{g_{\text{MeOH}}}{\text{mol}}}\times 100\;∼\;64\%$$

##### NH_3_ synthesis (gas-phase) from N_2_

The production efficiency for NH_3_ synthesis from N_2_ was evaluated using energy consumption reported by Hawtof et al.^44^^,^^49^ for the Haber-Bosch (H-B) process. H-B is the current industrial standard for NH_3_ production.

$$\text{Energy}\;\text{consumption}\;\text{in}\;H-B=9-11\frac{\text{kWh}}{{\text{kg}}_{\text{NH}3}}\text{.}$$

For a 10 kWh/kg_NH3_, the benchmark production efficiency can be evaluated as follows,

$$\text{Production}\;\text{efficiency}=\frac{\text{moles}\;\text{of}\;\text{fuel}\times {\text{LHV}}_{\text{fuel}}}{\text{total}\;\text{energy}\;\text{input}}\text{.}$$

Therefore,

$$\text{Production}\;\text{efficiency}\;\left[\%\right]=\frac{\text{moles}\;\text{of}\;{\text{NH}}_{3}\times \text{LH}V_{\text{NH}3}}{\text{total}\;\text{energy}\;\text{input}\;}\times 100$$

$$=\frac{319.6\frac{\text{kJ}}{\text{mol}}}{10\times \frac{3600}{1000}\times \frac{\text{kJ}}{g_{\text{NH}3}}\times 17\frac{g_{\text{NH}3}}{\text{mol}}}\times 100\;∼\;52\%$$

##### Electrochemical N_2_ reduction to NH_3_

The benchmark faradaic efficiency and energy consumption were taken from Hochman et al. and Wu et al.^51^^,^^52^ These values were reported as faradaic efficiency and energy consumption benchmarks based on techno-economic analysis of direct electrochemical N_2_ reduction to NH_3_,

Benchmarkfaradaicefficiency[%]=95%,

where faradaic efficiency = $\frac{\left(e^{-}\;\text{required}\;\text{for}\;a\;\text{mole}\;\text{of}\;NH_{3}\times NH_{3}\;\text{moles}\;\text{produced}\times \text{faradaic}\;\text{constant}\right)}{\text{charge}\;\text{transfer}}$,

$$\text{Energy}\;\text{consumption}=8.64\;\text{kWh}/kg_{\text{NH}3}\text{.}$$

$$\text{Production}\;\text{efficiency}=\frac{\text{moles}\;\text{of}\;\text{fuel}\times {\text{LHV}}_{\text{fuel}}}{\text{total}\;\text{energy}\;\text{input}}\text{.}$$

$$\text{Production}\;\text{efficiency}\;\left[\%\right]=\frac{\text{moles}\;\text{of}\;{\text{NH}}_{3}\times \text{LH}V_{\text{NH}3}}{\text{total}\;\text{energy}\;\text{input}\;}\times 100$$

$$=\frac{319.6\frac{\text{kJ}}{\text{mol}}}{8.64\times \frac{3600}{1000}\frac{\text{kJ}}{g_{\text{NH}3}}\times 17\frac{g_{\text{NH}3}}{\text{mol}}\;}\times 100\;∼\;60\%$$

##### Dry reforming of CH_4_ to CO

The production efficiency was based on performance criteria reported for plasma-assisted dry reforming of methane by Delikonstantis et al.

Energy consumption = 49.7 kWh/kmol_syngas_.

From the balanced equation of dry reforming of methane,

$$CH_{4}+CO_{2}\;\Rightarrow \;2\text{CO}+{2H}_{2}\text{,}$$

one mole of syngas is equivalent to a mole of CO and a mole of H_2_.

If the target fuel is CO, only half the total syngas yield that is coming from CO needs to be considered for the evaluation of production efficiency.

$$\text{Production}\;\text{efficiency}\;\left[\%\right]=\frac{\text{moles}\;\text{of}\;\text{fuel}\times {\text{LHV}}_{\text{fuel}}}{\text{total}\;\text{energy}\;\text{input}}\times 100$$

$$=\frac{\frac{1}{2}\times \text{moles}\;\text{of}\;\text{CO}\times \text{LH}V_{\text{CO}}}{\text{SEI}}\times 100$$

$$=\frac{1}{2}\times \frac{\text{LH}V_{\text{CO}}}{\text{Energy}\;\text{consumption}\;\text{per}\;\text{mole}\;\text{of}\;\text{syngas}\;\text{production}}\times 100$$

$$=\frac{1}{2}\times \frac{282.8}{49.7\times \frac{3600}{1000}}\times 100\;∼\;79\%$$

If H_2_ is the target fuel, only half the total syngas yield that is coming from H_2_ needs to be considered for the evaluation of production efficiency.

$$\text{Production}\;\text{efficiency}\;\left[\%\right]=\frac{\text{moles}\;\text{of}\;\text{fuel}\times {\text{LHV}}_{\text{fuel}}}{\text{total}\;\text{energy}\;\text{input}}\times 100$$

$$=\frac{1}{2}\times \frac{\text{LH}V_{H2}}{\text{Energy}\;\text{consumption}\;\text{per}\;\text{mole}\;\text{of}\;\text{syngas}\;\text{production}}\times 100$$

$$=\frac{1}{2}\times \frac{240}{49.7\times \frac{3600}{1000}}\times 100\;∼\;67\%$$

#### Uncertainty propagation for production efficiency

In this manuscript, we proposed a simpler definition (i.e., with fewer parameters) to evaluate the production efficiency of plasma-chemical processes. This section derives expressions for the uncertainty of both the conventional (Table 1) and our proposed definition (Equation 4). Here we are only considering plasma-driven chemical processes with a single target fuel.

##### Simplified definition of production efficiency (Equation 4)

We propose a production efficiency ($\eta_{f}$) metric that involves three parameters, including the lower heating value of the product (LHV_product_), product ratio of a desired target product ($Y_{\text{product}}$), and specific energy input (SEI),

$$\eta_{f}=\frac{\text{LH}V_{\text{product}}\;Y_{\text{product}}}{\text{SEI}}\text{.}$$

$$\eta_{f}=\frac{\text{LH}V_{\text{product}}\;{\hat{\dot{n}}}_{\text{product}}}{\bar{\text{power}}}\text{.}$$

In the case of a batch reactor,

$$\eta_{f}=\frac{\text{LH}V_{\text{product}}\;{\hat{n}}_{\text{product}}}{\text{total}\;\text{energy}}\text{,}$$

where $\hat{n}$ = number of moles.

Here, we chose flow reactor as an example to derive the relation for uncertainty propagation in production efficiency, however, replacing molar flow rates with number of moles and average power with total energy added would modify the expression for a batch reactor. $\eta_{f}=\frac{\text{LH}V_{\text{product}}\;{\hat{\dot{n}}}_{\text{product}}}{\bar{\text{power}}}\text{.}$

The measurement of $\eta_{f}$ involves two parameters (i.e., $\bar{\text{power}}$ and ${\hat{\dot{n}}}_{\text{product}}$) with measurement uncertainties that can vary from one facility to another. Each of these uncertainties contribute to the total uncertainty of the production efficiency for a plasma chemical process. Without considering propagation effects, efficiency metrics can have uncertainties that make them impractical to deploy.

If we assume the measurement of $\bar{\text{power}}$ and ${\hat{\dot{n}}}_{\text{product}}$ are independent, we can quantify the uncertainty in efficiency, $\delta \eta_{f}$ , by,

$$\delta \eta_{f}=\sqrt{{\left(\frac{\partial \eta_{f}}{\partial {\hat{\dot{n}}}_{\text{product}}}\delta {\hat{\dot{n}}}_{\text{product}}\right)}^{2}+{\left(\frac{\partial \eta_{f}}{\partial \bar{\text{power}}}\delta \bar{\text{power}}\right)}^{2}}\text{,}$$

$$\delta \eta_{f}=\sqrt{{\left(\frac{100\text{LH}V_{\text{product}}}{\bar{\text{power}}}\delta {\hat{\dot{n}}}_{\text{product}}\right)}^{2}+{\left(\frac{100\text{LH}V_{\text{product}}{\hat{\dot{n}}}_{\text{product}}}{{\bar{\text{power}}}^{2}}\delta \bar{\text{power}}\right)}^{2}}\text{,}$$

$$\delta \eta_{f}=\sqrt{{\left(\frac{100\text{LH}V_{\text{product}}{\hat{\dot{n}}}_{\text{product}}}{\bar{\text{power}}}\frac{\delta {\hat{\dot{n}}}_{\text{product}}}{{\hat{\dot{n}}}_{\text{product}}}\right)}^{2}+{\left(\frac{100\text{LH}V_{\text{product}}{\hat{\dot{n}}}_{\text{product}}}{\bar{\text{power}}}\frac{\delta \bar{\text{power}}}{\bar{\text{power}}}\right)}^{2}}\text{,}$$

$${\delta \eta }_{f}=\frac{100\text{LH}V_{\text{product}}{\hat{\dot{n}}}_{\text{product}}}{\bar{\text{power}}}\;\sqrt{{\left(\frac{\delta {\hat{\dot{n}}}_{\text{product}}}{{\hat{\dot{n}}}_{\text{product}}}\right)}^{2}+{\left(\frac{\delta \bar{\text{power}}}{\bar{\text{power}}}\right)}^{2}}=\eta_{f}\sqrt{{\left(\frac{\delta {\hat{\dot{n}}}_{\text{product}}}{{\hat{\dot{n}}}_{\text{product}}}\right)}^{2}+{\left(\frac{\delta \bar{\text{power}}}{\bar{\text{power}}}\right)}^{2}}\text{,}$$

$$\text{Uncertainty},\frac{\delta \eta_{f}}{\eta_{f}}=\sqrt{{\left(\frac{\delta {\hat{\dot{n}}}_{\text{product}}}{{\hat{\dot{n}}}_{\text{product}}}\right)}^{2}+{\left(\frac{\delta \bar{\text{power}}}{\bar{\text{power}}}\right)}^{2},}$$

where $\frac{\delta {\hat{\dot{n}}}_{\text{product}}}{{\hat{\dot{n}}}_{\text{product}}}$ is the molar relative uncertainty and $\frac{\delta \bar{\text{power}}}{\bar{\text{power}}}$ is the relative uncertainty in the measurement of average power.

##### Conventional definition of production efficiency (Table 1)

The conventional definition for production efficiency that is widely used in the literature includes 4 parameters, including the product ratio of the products, SEI and reactant conversions and reactant compositions. This introduces a larger measurement uncertainty compared to the proposed definition (Equation 4).

$$\eta_{f}=\frac{\sum_{j=1}^{\text{product}s}\text{LH}V_{j}Y_{j}}{\text{SEI}+\sum_{i=1}^{\text{reactants}}{\text{conversion}}_{i}\alpha_{i}\text{LH}V_{i}}\text{,}$$

In order to estimate the uncertainty propagation of the proposed definition, here we assume a reaction with one combustible reactant, one oxidizing reactant and one target fuel. For example, in dry reforming of methane, CH_4_ is the reactant with an LHV while CO_2_ is the oxidant. With these simplifications and substituting for reactant conversion, the expression for production efficiency becomes the following,

$$\eta_{f}=\frac{{\hat{\dot{n}}}_{\text{product}}{\text{LHV}}_{\text{product}}}{\bar{\text{power}}+\Delta {\hat{\dot{n}}}_{\text{reactant}}{\text{LHV}}_{\text{reactant}}}\text{.}$$

In the case of a batch reactor,

$$\eta_{f}=\frac{{\hat{n}}_{\text{product}}{\text{LHV}}_{\text{product}}}{\text{energy}\;\text{input}+\Delta {\hat{n}}_{\text{reactant}}{\text{LHV}}_{\text{reactant}}}\text{,}$$

where $\hat{n}$ = number of moles, $\Delta {\hat{n}}_{\text{reactant}}=\text{converted}\;\text{number}\;\text{of}\;\text{moles}\;\text{of}\;\text{the}\;\text{reactant.}$

Here, we chose flow reactor as an example to derive the relation for uncertainty propagation in production efficiency, however, replacing molar flow rates with number of moles and average power with total energy added would modify the expression for a batch reactor.

$$\eta_{f}=\frac{{\hat{\dot{n}}}_{\text{product}}\text{LH}V_{\text{product}}}{\bar{\text{power}}+\Delta {\hat{\dot{n}}}_{\text{reactant}}\text{LH}V_{\text{reactant}}}\text{.}$$

Assuming the measurement of power and moles of product are independent, we can once again quantify the uncertainty in efficiency, $\delta \eta_{f}$ , by,

$$\text{Uncertainty}\;\text{in}\;\text{efficiency},\delta \eta_{f}=\sqrt{{\left(\frac{\partial \eta_{f}}{\partial {\dot{n}}_{\text{product}}}\delta {\hat{\dot{n}}}_{\text{product}}\right)}^{2}+{\left(\frac{\partial \eta_{f}}{\partial \bar{\text{power}}}\delta \bar{\text{power}}\right)}^{2}+{\left(\frac{\partial \Delta {\hat{\dot{n}}}_{\text{reactant}}}{\partial \Delta {\hat{\dot{n}}}_{\text{reactant}}}\delta \Delta {\hat{\dot{n}}}_{\text{reactant}}\right)}^{2}}\text{,}$$

$$\delta \eta_{f}=\sqrt{{\left(\frac{\text{LH}V_{\text{product}}}{\bar{\text{power}}+\Delta {\hat{\dot{n}}}_{\text{reactant}}\text{LH}V_{\text{reactant}}}\delta {\hat{\dot{n}}}_{\text{product}}\right)}^{2}+{\left(\frac{\text{LH}V_{\text{product}}{\hat{\dot{n}}}_{\text{product}}}{{\left(\bar{\text{power}}+\Delta {\hat{\dot{n}}}_{\text{reactant}}\text{LH}V_{\text{reactant}}\right)}^{2}}\delta \bar{\text{power}}\right)}^{2}+{\left(\frac{\text{LH}V_{\text{product}}{\hat{\dot{n}}}_{\text{product}}\text{LH}V_{\text{reactant}}}{{\left(\bar{\text{power}}+\Delta {\hat{\dot{n}}}_{\text{reactant}}\times \text{LH}V_{\text{reactant}}\right)}^{2}}\delta \Delta {\hat{\dot{n}}}_{\text{reactant}}\right)}^{2}}\text{,}$$

$$\delta \eta_{f}=\sqrt{{\left(\frac{\text{LH}V_{\text{product}}{\hat{\dot{n}}}_{\text{product}}}{\bar{\text{power}}+\Delta {\hat{\dot{n}}}_{\text{reactant}}\text{LH}V_{\text{reactant}}}\frac{\delta {\hat{\dot{n}}}_{\text{product}}}{{\hat{\dot{n}}}_{\text{product}}}\right)}^{2}+{\left(\frac{\text{LH}V_{\text{product}}{\hat{\dot{n}}}_{\text{product}}}{\bar{\text{power}}+\Delta {\hat{\dot{n}}}_{\text{reactant}}\text{LH}V_{\text{reactant}}}\frac{\delta \bar{\text{power}}}{\bar{\text{power}}+\Delta {\hat{\dot{n}}}_{\text{reactant}}\text{LH}V_{\text{reactant}}}\right)}^{2}+{\left(\frac{\text{LH}V_{\text{product}}{\hat{\dot{n}}}_{\text{product}}}{\bar{\text{power}}+\Delta {\hat{\dot{n}}}_{\text{reactant}}\text{LH}V_{\text{reactant}}}\frac{\text{LH}V_{\text{reactant}}\delta \Delta {\hat{\dot{n}}}_{\text{reactant}}}{\bar{\text{power}}+\Delta {\hat{\dot{n}}}_{\text{reactant}}\text{LH}V_{\text{reactant}}}\right)}^{2}}\text{,}$$

$$\delta \eta_{f}=\eta_{f}\sqrt{{\left(\frac{\delta {\hat{\dot{n}}}_{\text{product}}}{{\hat{\dot{n}}}_{\text{product}}}\right)}^{2}+{\left(\frac{\delta \bar{\text{power}}}{\bar{\text{power}}+\Delta {\hat{\dot{n}}}_{\text{reactant}}\text{LH}V_{\text{reactant}}}\right)}^{2}+{\left(\frac{\text{LH}V_{\text{reactant}}\delta \Delta {\hat{\dot{n}}}_{\text{reactant}}}{\bar{\text{power}}+\Delta {\hat{\dot{n}}}_{\text{reactant}}\text{LH}V_{\text{reactant}}}\right)}^{2}}\text{.}$$

As the measurement of conversion introduces a measurement uncertainty in the initial and final number of moles of the reactant,

$$\Delta {\hat{\dot{n}}}_{\text{reactant}}={\left({\hat{\dot{n}}}_{\text{reactant}}\right)}_{\text{initial}}-{\left({\hat{\dot{n}}}_{\text{reactant}}\right)}_{\text{final}},\delta \Delta {\hat{\dot{n}}}_{\text{reactant}}=\sqrt{\delta {\hat{\dot{n}}}_{\text{reactant}}^{2}+\delta {\hat{\dot{n}}}_{\text{reactant}\;}^{2}}\text{,}$$

treating measurement errors of both reactants and products as the same as it is decided by the measuring instrument (i.e., gas chromatography instrument),

$$\delta \Delta {\hat{\dot{n}}}_{\text{reactant}}=\sqrt{\delta {\hat{\dot{n}}}_{\text{product}}^{2}+\delta {\hat{\dot{n}}}_{\text{product}}^{2}}=\sqrt{2}\;\delta {\hat{\dot{n}}}_{\text{product}}\text{,}$$

we can replace $\delta \Delta {\hat{\dot{n}}}_{\text{reactant}}$ with $\sqrt{2}\;\delta {\hat{\dot{n}}}_{\text{product}}$ to make two independent measurement variables $\delta \bar{\text{power}}$ and $\delta {\hat{\dot{n}}}_{\text{product}}$,

$$\delta \eta_{f}=\eta_{f}\sqrt{{\left(\frac{\delta {\hat{\dot{n}}}_{\text{product}}}{{\hat{\dot{n}}}_{\text{product}}}\right)}^{2}+{\left(\frac{\delta \bar{\text{power}}}{\bar{\text{power}}+\Delta {\hat{\dot{n}}}_{\text{reactant}}\text{LH}V_{\text{reactant}}}\right)}^{2}+{\left(\frac{\sqrt{2}\;\delta {\hat{\dot{n}}}_{\text{product}}\text{LH}V_{\text{reactant}}}{\bar{\text{power}}+\Delta {\hat{\dot{n}}}_{\text{reactant}}\text{LH}V_{\text{reactant}}}\right)}^{2}}\text{,}$$

$$\delta \eta_{f}=\eta_{f}\sqrt{{\left(\frac{\delta {\hat{\dot{n}}}_{\text{product}}}{{\hat{\dot{n}}}_{\text{product}}}\right)}^{2}+{\left(\frac{\delta \bar{\text{power}}}{\bar{\text{power}}+\Delta {\hat{\dot{n}}}_{\text{reactant}}\text{LH}V_{\text{reactant}}}\right)}^{2}+{\left(\frac{\sqrt{2}\;\delta {\hat{\dot{n}}}_{\text{product}}\text{LH}V_{\text{reactant}}}{\bar{\text{power}}+\Delta {\hat{\dot{n}}}_{\text{reactant}}\text{LH}V_{\text{reactant}}}\right)}^{2}}\text{,}$$

$$\delta \eta_{f}=\eta_{f}\sqrt{\begin{array}{c} {\left(\frac{\delta {\hat{\dot{n}}}_{\text{product}}}{{\hat{\dot{n}}}_{\text{product}}}\right)}^{2}+{\left(\frac{1}{\bar{\text{power}}+\Delta {\hat{\dot{n}}}_{\text{reactant}}\text{LH}V_{\text{reactant}}}\right)}^{2}\times \left({\left(\frac{\delta \bar{\text{power}}}{\bar{\text{power}}}\bar{\text{power}}\right)}^{2}+{\left(\text{LH}V_{\text{reactant}}\sqrt{2}\;\frac{\delta {\hat{\dot{n}}}_{\text{product}}}{{\hat{\dot{n}}}_{\text{product}}}{\hat{\dot{n}}}_{\text{product}}\right)}^{2}\right) \end{array}}\text{.}$$

If we call ${\left(\eta_{f}\right)}_{\text{conventional}}=\frac{\text{LH}V_{\text{product}}{\hat{\dot{n}}}_{\text{product}}}{\bar{\text{power}}+\Delta {\hat{\dot{n}}}_{\text{reactant}}\text{LH}V_{\text{reactant}}}$, we can simplify our definition using,

$${\left(\frac{1}{\bar{\text{power}}+\Delta {\hat{\dot{n}}}_{\text{reactant}}\text{LH}V_{\text{reactant}}}\right)}^{2}={\left(\frac{{\left(\eta_{f}\right)}_{\text{conventional}}}{\text{LH}V_{\text{product}}{\hat{\dot{n}}}_{\text{product}}}\right)}^{2}\text{.}$$

$$\delta \eta_{f}=\eta_{f}\sqrt{\begin{array}{c} {\left(\frac{\delta {\hat{\dot{n}}}_{\text{product}}}{{\hat{\dot{n}}}_{\text{product}}}\right)}^{2}+{\left(\frac{{\left(\eta_{f}\right)}_{\text{conventional}}}{\text{LH}V_{\text{product}}{\hat{\dot{n}}}_{\text{product}}}\right)}^{2}\times \left({\left(\frac{\delta \bar{\text{power}}}{\bar{\text{power}}}\bar{\text{power}}\right)}^{2}+{\left(\text{LH}V_{\text{reactant}}\sqrt{2}\;\frac{\delta {\hat{\dot{n}}}_{\text{product}}}{{\hat{\dot{n}}}_{\text{product}}}{\hat{\dot{n}}}_{\text{product}}\right)}^{2}\right) \end{array}}\text{,}$$

$$\delta \eta_{f}=\eta_{f}\sqrt{\begin{array}{c} {\left(\frac{\delta {\hat{\dot{n}}}_{\text{product}}}{{\hat{\dot{n}}}_{\text{product}}}\right)}^{2}+{\left(\frac{{\left(\eta_{f}\right)}_{\text{conventional}}\bar{\text{power}}}{\text{LH}V_{\text{product}}{\hat{\dot{n}}}_{\text{product}}}\right)}^{2}\\ \left({\left(\frac{\delta \bar{\text{power}}}{\bar{\text{power}}}\right)}^{2}+{\left(\frac{\text{LH}V_{\text{reactant}}}{\text{LH}V_{\text{product}}}\sqrt{2}\;\frac{\delta {\hat{\dot{n}}}_{\text{product}}}{{\hat{\dot{n}}}_{\text{product}}}\frac{\text{LH}V_{\text{product}}{\hat{\dot{n}}}_{\text{product}}}{\bar{\text{power}}}\right)}^{2}\right) \end{array}}\text{.}$$

Finally, we can utilize the proposed definition for the production efficiency, ${\left(\eta_{f}\right)}_{\text{proposed}}=\frac{\text{LH}V_{\text{product}}{\hat{\dot{n}}}_{\text{product}}}{\bar{\text{power}}}\text{,}$ to simply the uncertainty propagation of the conventional definition.

$$\delta \eta_{f}={\;\eta }_{f}\sqrt{\begin{array}{c} {\left(\frac{\delta {\hat{\dot{n}}}_{\text{product}}}{{\hat{\dot{n}}}_{\text{product}}}\right)}^{2}+{\left(\frac{{\left(\eta_{f}\right)}_{\text{conventional}}}{{\left(\eta_{f}\right)}_{\text{proposed}}}\right)}^{2}\left({\left(\frac{\delta \bar{\text{power}}}{\bar{\text{power}}}\right)}^{2}+{\left(\frac{\text{LH}V_{\text{reactant}}}{\text{LH}V_{\text{product}}}\sqrt{2}\;\frac{\delta {\hat{\dot{n}}}_{\text{product}}}{{\hat{\dot{n}}}_{\text{product}}}{\left(\eta_{f}\right)}_{\text{proposed}}\right)}^{2}\right) \end{array}}\text{.}$$

This expression can be further simplified by noting $\frac{{\left(\eta_{f}\right)}_{\text{conventional}}}{{\left(\eta_{f}\right)}_{\text{proposed}}}\;∼\;O\left(1\right)$ (Figure 5). The ${\left(\eta_{f}\right)}_{\text{proposed}}$ term can be replaced with a target performance metric for plasma chemical processes. Here, to show numerically the uncertainty propagation difference between the proposed definition and the conventional definition, we chose dry reforming of methane as an example. We chose the benchmark production efficiency of 79% (Figure 5A), as that is the range of benchmark efficiency values for plasma-driven dry reforming of methane.

$\text{Replacing}\;{\left(\eta_{f}\right)}_{\text{proposed}}=0.79\;\text{accordingly},$

$$\delta \eta_{f}=\eta_{f}\sqrt{\begin{array}{c} {\left(\frac{\delta {\hat{\dot{n}}}_{\text{product}}}{{\hat{\dot{n}}}_{\text{product}}}\right)}^{2}+\left({\left(\frac{\delta \bar{\text{power}}}{\bar{\text{power}}}\right)}^{2}+{\left(\frac{\text{LH}V_{\text{CH}4}}{\text{LH}V_{\text{product}}}\sqrt{2}\;\frac{\delta {\hat{\dot{n}}}_{\text{product}}}{{\hat{\dot{n}}}_{\text{product}}}\times 0.79\right)}^{2}\right) \end{array}}\text{.}$$

where $\frac{\delta {\hat{\dot{n}}}_{\text{product}}}{{\hat{\dot{n}}}_{\text{product}}}$ is the molar relative uncertainty and $\frac{\delta \bar{\text{power}}}{\bar{\text{power}}}$ is the relative uncertainty in the measurement of average power.

### Quantification and statistical analysis

This article does not present any new experimental data. In Figure 5, the comparison between the conventional and simplified definitions of production efficiency includes an estimation of standard deviations from their respective means. These deviations were calculated using 25 different datasets sourced from the literature.

## Footnotes

Supplemental information can be found online at https://doi.org/10.1016/j.isci.2025.112297.
