# Supplementary material for: What is “efficiency” in plasma chemical processes?
Source: iScience. 2025 Mar 29;28(5):112297. doi: 10.1016/j.isci.2025.112297 (PMC12032917; doi:10.1016/j.isci.2025.112297)
Supplement: Document S1. Figure S1 and Tables S1 and S2 [file mmc1.pdf]

iScience, Volume *28*

Supplemental information

What is “efficiency” in plasma chemical processes?

Charan R. Nallapareddy and Thomas C. Underwood

Supporting Information for

What is “efficiency” in plasma chemical processes?

Charan R. Nallapareddy and Thomas C. Underwood

Corresponding author: thomas.underwood@utexas.edu

The PDF file includes:

Supplementary Text
Table S1-S2
Figures S1
References 1- 52

Section 1. Definitions of reactor-based parameters used in the main text

The following definitions were used for conversion ($\text{Conversion}_{\text{reactant}}$), composition ($\alpha$), product ratio (Y), selectivity ($\text{Selectivity}^{A}_{\text{product}}$), yield ($\text{Yield}^{A}_{\text{product}}$), and specific energy input (SEI) that were discussed in the main text. Depending on the reactor configuration (i.e., flow or batch reactor mode), these relations change.

In a batch reactor mode,

$$\text{Conversion}_{\text{reactant}} = \frac{\hat{n}_{\text{reactant,initial}} - \hat{n}_{\text{reactant,final}}}{\hat{n}_{\text{reactant,initial}}} = \frac{\Delta\hat{n}_{\text{reactant}}}{\hat{n}_{\text{reactant,initial}}},$$

where $\hat{n}$ = number of moles.

$$\text{Total conversion} = \sum_{i=1}^{\text{reactants}} \alpha_i \text{ conversion}_i,$$

$$\text{where composition, } \alpha_i = \frac{\hat{n}_{i,\text{initial}}}{\sum_{i=1}^{\text{reactants}} \hat{n}_{i,\text{initial}}}.$$

$$\text{SEI} = \frac{\text{total energy}}{\sum_{i=1}^{\text{reactants}} \hat{n}_{i,\text{initial}}}.$$

$$\text{Product ratio, Y} = \frac{\hat{n}_{\text{product}}}{\sum_{i=1}^{\text{reactants}} \hat{n}_{i,\text{initial}}}.$$

Other parameters that are used commonly to denote the performance of reactors by tracking the conversion of atomic species A include,

$$\text{Selectivity}^{A}_{\text{product}} = \frac{\beta^{A}_{\text{product}}\hat{n}_{\text{product}}}{\sum_{i=1}^{\text{reactants}} \beta^{A}_{i}\Delta\hat{n}_i},$$

$$\text{Yield}^{A}_{\text{product}} = \frac{\beta^{A}_{\text{product}}\hat{n}_{\text{product}}}{\sum_{i=1}^{\text{reactants}} \beta^{A}_{i}\hat{n}_{i,\text{initial}}},$$

where $\beta^{A}_{\text{product}}$ and $\beta^{A}_{\text{reactant}}$ are the number of atoms A per product and reactant respectively, and atom A can be any atom present in the reaction under consideration.

In a flow reactor mode,

$$\text{Conversion}_{\text{reactant}} = \frac{\dot{\hat{n}}_{\text{reactant,initial}} - \dot{\hat{n}}_{\text{reactant,final}}}{\dot{\hat{n}}_{\text{reactant,initial}}} = \frac{\Delta\dot{\hat{n}}_{\text{reactant}}}{\dot{\hat{n}}_{\text{reactant,initial}}},$$

where $\dot{\hat{n}}$ = molar flow rate.

$$\text{Total conversion [\%]} = \sum_{i=1}^{\text{reactants}} \alpha_i \,\text{conversion}_i,$$

$$\text{where composition, } \alpha_i = \frac{\hat{\dot{n}}_{i,\text{initial}}}{\sum_{i=1}^{\text{reactants}} \hat{\dot{n}}_{i,\text{initial}}}.$$

$$\text{SEI} = \frac{\overline{\text{power}}}{\sum_{i=1}^{\text{reactants}} \hat{\dot{n}}_{i,\text{initial}}}, \text{ where } \overline{\text{power}} \text{ is average power.}$$

$$\text{Product ratio, Y} = \frac{\hat{\dot{n}}_{\text{product}}}{\sum_{i=1}^{\text{reactants}} \hat{\dot{n}}_{i,\text{initial}}}.$$

Other parameters that are used commonly to denote the performance of reactors by tracking the conversion of atomic species A include,

$$\text{Selectivity}_{\text{product}}^{A} = \frac{\beta_{\text{product}}^{A} \hat{\dot{n}}_{\text{product}}}{\sum_{i=1}^{\text{reactants}} \beta_i^{A} \Delta\hat{\dot{n}}_i},$$

$$\text{Yield}_{\text{product}}^{A} = \frac{\beta_{\text{product}}^{A} \hat{\dot{n}}_{\text{product}}}{\sum_{i=1}^{\text{reactants}} \beta_i^{A} \hat{\dot{n}}_{i,\text{initial}}}.$$

Section 2. Nomenclature and definitions of conversion efficiency

Table S1. The diverse nomenclature and definitions of conversion efficiency with references that highlight its usage in the literature.

Inconsistent Names for the Metric	Inconsistent Definitions for the Metric	Source
Energy efficiency	$\eta_c = \frac{\Delta H_r\left(\frac{kJ}{mol}\right) \times N_{product}(mol)}{\text{Power Input (W)}}$	1
Energy efficiency	$\eta_c = \frac{F_{out}\left(\frac{mol}{s}\right) H_{out}\left(\frac{kJ}{mol}\right) \text{-} F_{in}\left(\frac{mol}{s}\right) H_{in}\left(\frac{kJ}{mol}\right)}{\text{Power Input (W)}}$	2
Energy efficiency/Conversion efficiency/Energy cost	$\eta_c = \frac{\Delta H_r\left(\frac{kJ}{mol}\right) \times \text{Conversion (\%)}}{\text{Specific Energy Input}\left(\frac{kJ}{mol}\right)}$<br>where conversion (%) = $\sum_{reactants} \text{Conversion}_{reactant}\ [\%] \times \text{mole fraction}_{reactant}$	3–11
Chemical energy efficiency	$\eta_c = \frac{\Delta H_r\left(\frac{kJ}{mol}\right)}{\text{Specific Energy Input}\left(\frac{kJ}{mol}\right)}$	12–15
Energy efficiency	$\eta_c = \frac{n_{fuel}(mol) \times \Delta H_f\left(\frac{kJ}{mol}\right) - \sum_{reactants} \Delta n_{converted}(mol) \times \Delta H_f\left(\frac{kJ}{mol}\right)}{\text{Energy Input (kJ)}} \times 100$	16
Conversion efficiency	$\eta_c = \frac{\text{mass of converted reactant (mg)}}{\text{Energy Input (J)}}$	17,18
Plasma efficiency	$\eta_c = \frac{\text{Conversion (\%)}}{\text{Power Input (W)}}$	19
Energy efficiency for gas conversion/Conversion ability/ Plasma efficiency	$\eta_c = \frac{\sum_{reactants} \Delta \dot{n}_{converted}\left(\frac{mol}{s}\right)}{\text{Power (W)}}$	20–41

The highlighted definitions were used in the manuscript to compare definitions of conversion efficiency (Fig. 3) based on their usage in the literature.

Section 3. Benchmark values for efficiency metrics

Table S2. Benchmark performance for various plasma-chemical processes using the framework.

Plasma Chemical Process	Benchmark	Plasma Chemical Process	Benchmark(s)	
CO_2 splitting	$\eta_{c,benchmark}$ ~ 60%	*NH_3 synthesis*	$\eta_{f,benchmark}$ ~ 52%	
CH_4 partial oxidation	$\eta_{f,benchmark}$ ~ 64%	*CH_4 dry reforming*	$\eta_{f,benchmark}$ ~ 79%	
CH_4 pyrolysis	$\eta_{f,benchmark}$ ~ 85%	*Electrochemical N_2 reduction*	$\eta_{f,benchmark}$ ~ 60%	FE ~ 95%

Section 4. Data mining using Web of Science

The Web of Science public data was used as a source to obtain statistics on plasma related literature. The following portal was used to access the data from the Web of Science: https://www.webofscience.com/wos/woscc/basic-search.

The following advanced search commands were used to obtain Fig. 1A & 1B in the main text,

1. Total publications on plasma chemistry

   Keywords: Plasma AND reforming OR chemistry OR conversion
   Year range: 1980 – 2023

2. Plasma chemistry papers with the word "efficiency"

   Keywords: Plasma AND efficiency AND reforming OR chemistry OR conversion
   Year range: 1980 – 2023

3. Total number of "plasma reforming + efficiency" papers with "conversion efficiency"

   Keywords: Plasma AND reforming OR chemistry OR conversion AND "conversion efficiency"
   Year range: 1980 – 2023

4. Total number of "plasma reforming + efficiency" papers with "fuel production efficiency" or equivalent

   Keywords: Plasma AND reforming OR chemistry OR conversion AND "fuel efficiency" OR "fuel production efficiency" OR "Cold gas efficiency" OR "Energy conversion efficiency"
   Year range: 1980 – 2023

5. Total number of "plasma reforming + efficiency" papers with "energy efficiency"

   Keywords: Plasma AND reforming OR chemistry OR conversion AND "Energy efficiency"
   Year range: 1980 – 2023

6. Total number of "plasma reforming + efficiency" papers with "faradaic efficiency"

   Keywords: Plasma AND reforming OR chemistry OR conversion AND "faradaic efficiency" OR "faraday efficiency"
   Year range: 1980 – 2023

Section 5. Derivation of the simplified definition for production efficiency

The conventional definition for production efficiency that is widely used in the literature includes 4 parameters, the fuel ratio (Y), SEI, reactant conversion, and reactant composition (α).

$$\eta_{f,\ conventional} = \frac{\sum_{j=1}^{fuels} LHV_j Y_j}{SEI + \sum_{i=1}^{reactants} conversion_i \alpha_i LHV_i},$$

$$\Rightarrow \eta_{f,\ conventional} = \frac{\sum_{j=1}^{fuels} \hat{n}_j LHV_j}{\overline{power} + \sum_{i=1}^{reactants} \Delta\hat{n}_i LHV_i}.$$

For most common plasma chemical reactions (i.e., one target fuel/product, one combustible reactant and/or one non-combustible reactant) like dry reforming of methane, partial oxidation of methane, and ammonia synthesis,

$$\eta_{f,\ conventional} = \frac{LHV_{fuel} Y_{fuel}}{\overline{power} + \Delta\hat{n}\ \alpha\ LHV\ |_{reactant}}.$$

The conversion of non-combustible reactants, like O_2 and CO_2, do not contribute as $LHV_{non\text{-}combustible\ reactant} = 0$.

$$\Rightarrow \eta_{f,\ conventional} = \frac{LHV_{fuel} \hat{n}_{fuel}}{\overline{power} + \Delta\hat{n}_{reactant}\ LHV_{reactant}}.$$

Now, to simplify this relation further, we establish a relation between $\overline{power}$ and $\Delta\hat{n}_{reactant}$ by considering a simple uni-molecular reactant reaction,

$$A \xrightarrow{k} Products$$

$$\frac{d\hat{n}_A}{dt} = -k\hat{n}_A,$$

$$Conversion = \frac{\Delta\hat{n}_A}{\hat{n}_A} = (1 - \exp(-kt)). \qquad (A)$$

Now, at lower conversions or when kt<< 1 (i.e., shorter total reaction times), this relation can be further simplified. Using a Taylor expansion,

$$Conversion \approx 1-(1 - kt) = kt \propto k\times Energy\ Input \propto k\times SEI \propto k\times\overline{power}.$$

$$\Rightarrow Conversion = \frac{\Delta\hat{n}}{\hat{n}} \propto k\times\overline{power} \Rightarrow \Delta\hat{n} \propto \overline{power}.$$

Therefore, $\Delta\hat{n}_{reactant}$ is a linear function of average input power,

$$\Rightarrow \overline{power} + \Delta\hat{n}_{reactant} LHV_{reactant} = \overline{power} + f(\overline{power}) \propto \overline{power}.$$

$$\Rightarrow \ \eta_{f,\ proposed} = \frac{LHV_{product}\hat{\dot{n}}_{product}}{\overline{power}}.$$

At high conversion, Eq. A shows that the rate of change of conversion plateaus with average input power.

$$\Rightarrow \ \overline{power} > \Delta\hat{\dot{n}}_{reactant} LHV_{reactant} \ \Rightarrow \ \overline{power} + \Delta\hat{\dot{n}}_{reactant} LHV_{reactant} \sim \overline{power}.$$

$$\Rightarrow \ \eta_{f,\ proposed} = \frac{LHV_{product}\hat{\dot{n}}_{product}}{\overline{power}}.$$

This is the proposed definition for production efficiency that we used in the main text.

Section 6. Equivalence between η_c and η_f for type I reactions like CO_2 splitting

In this section, we show that conversion efficiency, η_c, and production efficiency, η_f, are equivalent for type I reactions (i.e., endothermic reactions with a fixed or known product distribution). The general definition for production efficiency is,

$$\eta_{f,\ conventional} = \frac{\sum_{j=1}^{fuels} LHV_j Y_j}{SEI + \sum_{i=1}^{reactants} conversion_i \alpha_i LHV_i}.$$

$$\Rightarrow \eta_f = \frac{\sum_{j=1}^{fuels} \hat{n}_j LHV_j}{\overline{power} + \sum_{i=1}^{reactants} \Delta\hat{n}_i LHV_i}.$$

For type I reactions (i.e., endothermic reactions with a known heat of reaction, $\Delta\widehat{H}_r$),

$$\Delta\widehat{H}_r \sum_{i=1}^{reactants} \Delta\hat{n}_i = \sum_{j=1}^{products} \hat{n}_j\, \Delta\widehat{H}_{f,j} - \sum_{i=1}^{reactants} \hat{n}_i\, \Delta\widehat{H}_{f,i},$$

where $\Delta\widehat{H}_f$ is the heat of formation of the species under consideration.

However, $\Delta\widehat{H}_r$can also be related to LHV of the species,

$$\Delta\widehat{H}_r \sum_{i=1}^{reactants} \Delta\hat{n}_i = \sum_{j=1}^{products} \hat{n}_j\, LHV_j - \sum_{i=1}^{reactants} \hat{n}_i\, LHV_i\ .$$

$$\Rightarrow \sum_{j=1}^{products} \hat{n}_j\, LHV_j = \sum_{i=1}^{reactants} \hat{n}_i\, LHV_i + \Delta\widehat{H}_r \sum_{i=1}^{reactants} \Delta\hat{n}_i\ .$$

Let's consider an example of a type I reaction like CO_2 splitting with no reactant LHV,

$$CO_2 \rightarrow CO + \frac{1}{2} O_2$$

For this reaction, $\sum_{i=1}^{reactants} \Delta\hat{n}_i\, LHV_i = \Delta\hat{n}_{CO2} LHV_{CO2} = 0$ and $\sum_{i=1}^{reactants} \hat{n}_i\, LHV_i = \hat{n}_{CO2} LHV_{CO2} = 0$ because $LHV_{CO2} = 0$.

$$\Rightarrow \eta_f = \frac{\Delta\widehat{H}_r \sum_{i=1}^{reactants} \Delta\hat{n}_i}{\overline{power}} = \frac{\Delta\widehat{H}_r \sum_{i=1}^{reactants} \left(\frac{\Delta\hat{n}_i}{\sum_{i=1}^{reactants} \hat{n}_{i,initial}} \right)}{\frac{\overline{power}}{\sum_{i=1}^{reactants} \hat{n}_{i,initial}}}.$$

Here $\frac{\overline{power}}{\sum_{i=1}^{reactants} \hat{n}_{i,initial}} = SEI$. Therefore,

$$\Rightarrow \eta_f = \frac{\Delta\hat{H}_r \sum_{i=1}^{reactants}\left(\frac{\Delta\hat{\dot{n}}_i}{\sum_{i=1}^{reactants}\hat{\dot{n}}_{i,initial}}\right)}{SEI},$$

$$= \frac{\Delta\hat{H}_r \sum_{i=1}^{reactants}\left(\frac{\Delta\hat{\dot{n}}_i}{\hat{\dot{n}}_{i,initial\ reactant}}\right)\left(\frac{\hat{\dot{n}}_{i,initial\ reactant}}{\sum_{i=1}^{reactants}\hat{\dot{n}}_{i,initial}}\right)}{SEI}.$$

Since $\text{conversion}_i = \frac{\Delta\hat{\dot{n}}_i}{\hat{\dot{n}}_{i,initial\ reactant}}$, and mole fraction $(\alpha_i) = \frac{\hat{\dot{n}}_{i,initial\ reactant}}{\sum_{i=1}^{reactants}\hat{\dot{n}}_{i,initial}}$,

$$\eta_f = \frac{\Delta\hat{H}_r \sum_{i=1}^{reactants} \text{conversion}_i\ \alpha_i}{SEI} = \eta_c. \quad (A)$$

This establishes the equivalence between η_c and η_f because eq. (A) is also the definition for conversion efficiency, η_c (table 1, main text).

Section 7. Usage of various definitions in the community for a given type of reaction

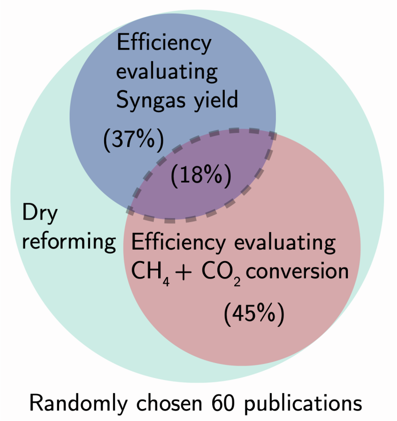

Figure S1. A survey of literature on plasma-driven dry reforming of methane showing reporting of different efficiency metrics (i.e., production efficiency, conversion efficiency, or both).

Since conversion efficiency requires ΔH_r evaluation, which in turn depends on unknown product distribution, multi-reactant plasma-chemical endothermic processes can be uniquely evaluated using production efficiency alone. A total of 60 randomly chosen publications were surveyed (a limited number due to diverse nomenclature and definitions used in the literature) from the web of science database for the reporting of these metrics, of which 45% reports conversion efficiency alone. The diameter of each shaded region is proportional to the number of publications.

Section 8. Using the efficiency framework for abatement processes

Abatement processes can be both endothermic (e.g., CO_2 splitting) and exothermic (i.e., N_2O abatement). Just like any other exothermic and endothermic processes and their sub-classes (i.e., type I, II, III), abatement processes can also make sure of this universal framework. As long as $\Delta\widehat{H}_r$ of the reaction is known and is positive, conversion efficiency can be used as the performance metric. If $\Delta\widehat{H}_r$ is unknown or negative, production efficiency must be used. The absence of a target product or fuel in abatement processes does not preclude using production efficiency as a performance metric. By leveraging potential products with LHV (e.g., products with non-zero LHV like N_2 in the case of N_2O conversion), production efficiency can be used to assess abatement processes. Even though the primary goal is not generating these products, production efficiency serves as a proxy for evaluating abatement performance.

References

1. Nozaki, T., and Okazaki, K. (2013). Non-thermal plasma catalysis of methane: Principles, energy efficiency, and applications. Catal Today *211*, 29–38. https://doi.org/10.1016/J.CATTOD.2013.04.002.
2. Ravasio, S., and Cavallotti, C. (2012). Analysis of reactivity and energy efficiency of methane conversion through non thermal plasmas. Chem Eng Sci *84*, 580–590. https://doi.org/10.1016/J.CES.2012.09.012.
3. Snoeckx, R., and Bogaerts, A. (2017). Plasma technology – a novel solution for CO2 conversion? Chem Soc Rev *46*, 5805–5863. https://doi.org/10.1039/C6CS00066E.
4. Vermeiren, V., and Bogaerts, A. (2019). Improving the Energy Efficiency of CO2 Conversion in Nonequilibrium Plasmas through Pulsing. Journal of Physical Chemistry C *123*, 17650–17665. https://doi.org/10.1021/ACS.JPCC.9B02362.
5. Adrianto, D., Sheng, Z., Nozaki, T. (2020). Mechanistic study on nonthermal plasma conversion of CO2. ijpest.securesite.jp *14*, 1003–1012. https://doi.org/10.34343/ijpest.2020.14.e01003.
6. Kim, H., Song, S., Tom, C.P., and Xie, F. (2020). Carbon dioxide conversion in an atmospheric pressure microwave plasma reactor: Improving efficiencies by enhancing afterglow quenching. Journal of CO2 Utilization *37*, 240–247. https://doi.org/10.1016/J.JCOU.2019.12.011.
7. Berthelot, A., and Bogaerts, A. (2018). Pinpointing energy losses in CO2 plasmas – Effect on CO2 conversion. Journal of CO2 Utilization *24*, 479–499. https://doi.org/10.1016/J.JCOU.2018.02.011.
8. Wolf, A.J., Peeters, F.J.J., Groen, P.W.C., Bongers, W.A., and Van De Sanden, M.C.M. (2020). CO2 Conversion in Nonuniform Discharges: Disentangling Dissociation and Recombination Mechanisms. Journal of Physical Chemistry C *124*, 16806–16819. https://doi.org/10.1021/ACS.JPCC.0C03637.
9. Slaets, J., Aghaei, M., Ceulemans, S., Van Alphen, S., and Bogaerts, A. (2020). CO2 and CH4 conversion in “real” gas mixtures in a gliding arc plasmatron: how do N2 and O2 affect the performance? Green Chemistry *22*, 1366–1377. https://doi.org/10.1039/C9GC03743H.
10. Snoeckx, R., Zeng, Y.X., Tu, X., and Bogaerts, A. (2015). Plasma-based dry reforming: improving the conversion and energy efficiency in a dielectric barrier discharge. RSC Adv *5*, 29799–29808. https://doi.org/10.1039/C5RA01100K.
11. Uytdenhouwen, Y., Bal, K.M., Neyts, E.C., Meynen, V., Cool, P., and Bogaerts, A. (2021). On the kinetics and equilibria of plasma-based dry reforming of methane. Chemical Engineering Journal *405*, 126630. https://doi.org/10.1016/J.CEJ.2020.126630.
12. Ghorbanzadeh, A.M., Lotfalipour, R., and Rezaei, S. (2009). Carbon dioxide reforming of methane at near room temperature in low energy pulsed plasma. Int J Hydrogen Energy *34*, 293–298. https://doi.org/10.1016/J.IJHYDENE.2008.10.056.
13. Chen, G., Georgieva, V., Godfroid, T., Snyders, R., and Delplancke-Ogletree, M.P. (2016). Plasma assisted catalytic decomposition of CO2. Appl Catal B *190*, 115–124. https://doi.org/10.1016/J.APCATB.2016.03.009.
14. Bak, M., Im, S., Cappelli, M. (2015). Nanosecond-pulsed discharge plasma splitting of carbon dioxide. ieeexplore.ieee.org, IEEE Transactions on Plasma Science, 2015•ieeexplore.ieee.org.

15. Iwarere, S.A., Rohani, V.J., Ramjugernath, D., and Fulcheri, L. (2015). Dry reforming of methane in a tip–tip arc discharge reactor at very high pressure. Int J Hydrogen Energy *40*, 3388–3401. https://doi.org/10.1016/J.IJHYDENE.2015.01.005.
16. Li, M.W., Xu, G.H., Tian, Y.L., Chen, L., and Fu, H.F. (2004). Carbon Dioxide Reforming of Methane Using DC Corona Discharge Plasma Reaction. Journal of Physical Chemistry A *108*, 1687–1693. https://doi.org/10.1021/JP037008Q.
17. Zhang, X., and Cha, M.S. (2015). Partial oxidation of methane in a temperature-controlled dielectric barrier discharge reactor. Proceedings of the Combustion Institute *35*, 3447–3454. https://doi.org/10.1016/J.PROCI.2014.05.089.
18. Zhang, X., and Suk Cha, M. (2013). Electron-induced dry reforming of methane in a temperature-controlled dielectric barrier discharge reactor. J Phys D Appl Phys *46*, 415205. https://doi.org/10.1088/0022-3727/46/41/415205.
19. Goujard, V., Tatibouët, J.M., and Batiot-Dupeyrat, C. (2009). Use of a non-thermal plasma for the production of synthesis gas from biogas. Appl Catal A Gen *353*, 228–235. https://doi.org/10.1016/J.APCATA.2008.10.050.
20. Yao, S.L., Ouyang, F., Nakayama, A., Suzuki, E., Okumoto, M., and Mizuno, A. (2000). Oxidative coupling and reforming of methane with carbon dioxide using a high-frequency pulsed plasma1. Energy and Fuels *14*, 910–914. https://doi.org/10.1021/EF000016A.
21. Ray, D., Reddy, P.M.K., and Subrahmanyam, C. (2018). Ni-Mn/γ-Al2O3 assisted plasma dry reforming of methane. Catal Today *309*, 212–218. https://doi.org/10.1016/J.CATTOD.2017.07.003.
22. Andersen, J.A., Christensen, J.M., Østberg, M., Bogaerts, A., and Jensen, A.D. (2020). Plasma-catalytic dry reforming of methane: Screening of catalytic materials in a coaxial packed-bed DBD reactor. Chemical Engineering Journal *397*, 125519. https://doi.org/10.1016/J.CEJ.2020.125519.
23. Li, D., Li, X., Bai, M., Tao, X., Shang, S., Dai, X., and Yin, Y. (2009). CO2 reforming of CH4 by atmospheric pressure glow discharge plasma: A high conversion ability. Int J Hydrogen Energy *34*, 308–313. https://doi.org/10.1016/J.IJHYDENE.2008.10.053.
24. Vakili, R., Gholami, R., Stere, C.E., Chansai, S., Chen, H., Holmes, S.M., Jiao, Y., Hardacre, C., and Fan, X. (2020). Plasma-assisted catalytic dry reforming of methane (DRM) over metal-organic frameworks (MOFs)-based catalysts. Appl Catal B *260*, 118195. https://doi.org/10.1016/J.APCATB.2019.118195.
25. Khoja, A.H., Tahir, M., and Amin, N.A.S. (2018). Cold plasma dielectric barrier discharge reactor for dry reforming of methane over Ni/ɤ-Al2O3-MgO nanocomposite. Fuel Processing Technology *178*, 166–179. https://doi.org/10.1016/J.FUPROC.2018.05.030.
26. Wang, H., Zhao, B., Qin, L., Wang, Y., Yu, F., and Han, J. (2020). Non-thermal plasma-enhanced dry reforming of methane and CO2 over Ce-promoted Ni/C catalysts. Molecular Catalysis *485*, 110821. https://doi.org/10.1016/J.MCAT.2020.110821.
27. Wang, H., Han, J., Bo, Z., Qin, L., Wang, Y., and Yu, F. (2019). Non-thermal plasma enhanced dry reforming of CH4 with CO2 over activated carbon supported Ni catalysts. Molecular Catalysis *475*, 110486. https://doi.org/10.1016/J.MCAT.2019.110486.
28. Khoja, A.H., Tahir, M., and Saidina Amin, N.A. (2019). Process optimization of DBD plasma dry reforming of methane over Ni/La2O3MgAl2O4 using multiple response surface methodology. Int J Hydrogen Energy *44*, 11774–11787. https://doi.org/10.1016/J.IJHYDENE.2019.03.059.

29. Shapoval, V., Marotta, E., Ceretta, C., Konjevič, N., Ivkovič, M., Schiorlin, M., and Paradisi, C. (2014). Development and Testing of a Self-Triggered Spark Reactor for Plasma Driven Dry Reforming of Methane. Plasma Processes and Polymers *11*, 787–797. https://doi.org/10.1002/PPAP.201400007.
30. Liu, L., Wang, Q., Song, J., Yang, X., and Sun, Y. (2018). Dry reforming of model biomass pyrolysis products to syngas by dielectric barrier discharge plasma. Int J Hydrogen Energy *43*, 10281–10293. https://doi.org/10.1016/J.IJHYDENE.2018.04.112.
31. Tao, X., Qi, F., Yin, Y., and Dai, X. (2008). CO2 reforming of CH4 by combination of thermal plasma and catalyst. Int J Hydrogen Energy *33*, 1262–1265. https://doi.org/10.1016/J.IJHYDENE.2007.12.057.
32. Khoja, A.H., Tahir, M., and Amin, N.A.S. (2019). Recent developments in non-thermal catalytic DBD plasma reactor for dry reforming of methane. Energy Convers Manag *183*, 529–560. https://doi.org/10.1016/J.ENCONMAN.2018.12.112.
33. Khoja, A.H., Tahir, M., and Amin, N.A.S. (2017). Dry reforming of methane using different dielectric materials and DBD plasma reactor configurations. Energy Convers Manag *144*, 262–274. https://doi.org/10.1016/J.ENCONMAN.2017.04.057.
34. Shapoval, V., and Marotta, E. (2015). Investigation on Plasma-Driven Methane Dry Reforming in a Self-Triggered Spark Reactor. Plasma Processes and Polymers *12*, 808–816. https://doi.org/10.1002/PPAP.201400177.
35. Diao, Y., Wang, H., Chen, B., Zhang, X., and Shi, C. (2023). Modulating morphology and textural properties of Al2O3 for supported Ni catalysts toward plasma-assisted dry reforming of methane. Appl Catal B *330*, 122573. https://doi.org/10.1016/J.APCATB.2023.122573.
36. Zeng, Y.X., Wang, L., Wu, C.F., Wang, J.Q., Shen, B.X., and Tu, X. (2018). Low temperature reforming of biogas over K-, Mg- and Ce-promoted Ni/Al2O3 catalysts for the production of hydrogen rich syngas: Understanding the plasma-catalytic synergy. Appl Catal B *224*, 469–478. https://doi.org/10.1016/J.APCATB.2017.10.017.
37. Kwon, H., Kim, T., and Song, S. (2023). Dry reforming of methane in a rotating gliding arc plasma: Improving efficiency and syngas cost by quenching product gas. Journal of CO2 Utilization *70*, 102448. https://doi.org/10.1016/J.JCOU.2023.102448.
38. Zhu, M., Zhong, A., Dai, D., Wang, Q., Shao, T., and Ostrikov, K. (2022). Surface-induced gas-phase redistribution effects in plasma-catalytic dry reforming of methane: numerical investigation by fluid modeling. J Phys D Appl Phys *55*, 355201. https://doi.org/10.1088/1361-6463/AC74F7.
39. Zhu, M., Zhong, A., Dai, D., -, al, Pan, J., Chen, T., Gao, Y., Cimerman, R., Raková, D., Hensel -, K., et al. (2011). Dry reforming of methane over a Ni/Al2O3 catalyst in a coaxial dielectric barrier discharge reactor. J Phys D Appl Phys *44*, 274007. https://doi.org/10.1088/0022-3727/44/27/274007.
40. Tu, X., and Whitehead, J.C. (2012). Plasma-catalytic dry reforming of methane in an atmospheric dielectric barrier discharge: Understanding the synergistic effect at low temperature. Appl Catal B *125*, 439–448. https://doi.org/10.1016/J.APCATB.2012.06.006.
41. Tu, X., and Whitehead, J.C. (2014). Plasma dry reforming of methane in an atmospheric pressure AC gliding arc discharge: Co-generation of syngas and carbon nanomaterials. Int J Hydrogen Energy *39*, 9658–9669. https://doi.org/10.1016/J.IJHYDENE.2014.04.073.

42. Bogaerts, A., and Centi, G. (2020). Plasma Technology for CO2 Conversion: A Personal Perspective on Prospects and Gaps. Front Energy Res *8*, 533985. https://doi.org/10.3389/FENRG.2020.00111/BIBTEX.
43. Osorio-Tejada, J., van't Veer, K., Long, N.V.D., Tran, N.N., Fulcheri, L., Patil, B.S., Bogaerts, A., and Hessel, V. (2022). Sustainability analysis of methane-to-hydrogen-to-ammonia conversion by integration of high-temperature plasma and non-thermal plasma processes. Energy Convers Manag *269*, 116095. https://doi.org/10.1016/J.ENCONMAN.2022.116095.
44. Lange, J.P. (1997). Perspectives for Manufacturing Methanol at Fuel Value. Ind Eng Chem Res *36*, 4282–4290. https://doi.org/10.1021/IE9607762.
45. Hawtof, R., Ghosh, S., Guarr, E., Xu, C., Sankaran, R.M., and Renner, J.N. (2019). Catalyst-free, highly selective synthesis of ammonia from nitrogen and water by a plasma electrolytic system. Asian Journal of Chemistry *31*, 5778–5789. https://doi.org/10.1126/SCIADV.AAT5778.
46. Hong, J., Aramesh, M., Shimoni, O., Seo, D.H., Yick, S., Greig, A., Charles, C., Prawer, S., and Murphy, A.B. (2016). Plasma Catalytic Synthesis of Ammonia Using Functionalized-Carbon Coatings in an Atmospheric-Pressure Non-equilibrium Discharge. Plasma Chemistry and Plasma Processing *36*, 917–940. https://doi.org/10.1007/S11090-016-9711-8.
47. Delikonstantis, E., Scapinello, M., and Stefanidis, G.D. (2017). Investigating the Plasma-Assisted and Thermal Catalytic Dry Methane Reforming for Syngas Production: Process Design, Simulation and Evaluation. Energies 2017, Vol. 10, Page 1429 *10*, 1429. https://doi.org/10.3390/EN10091429.
48. Hochman, G., Goldman, A.S., Felder, F.A., Mayer, J.M., Miller, A.J.M., Holland, P.L., Goldman, L.A., Manocha, P., Song, Z., and Aleti, S. (2020). Potential Economic Feasibility of Direct Electrochemical Nitrogen Reduction as a Route to Ammonia. ACS Sustain Chem Eng *8*, 8938–8948. https://doi.org/10.1021/ACSSUSCHEMENG.0C01206.
49. Wu, A., Yang, J., Xu, B., Wu, X.Y., Wang, Y., Lv, X., Ma, Y., Xu, A., Zheng, J., Tan, Q., et al. (2021). Direct ammonia synthesis from the air via gliding arc plasma integrated with single atom electrocatalysis. Appl Catal B *299*, 120667. https://doi.org/10.1016/J.APCATB.2021.120667.
50. Producing industrial hydrogen from renewable energy – Analysis - IEA https://www.iea.org/commentaries/producing-industrial-hydrogen-from-renewable-energy.
51. Nallapareddy, C.R., and Underwood, T.C. (2024). Tailoring Vibrational Excitation Pathways for High-Yield Oxidation of Methane to Methanol. ACS Sustain Chem Eng *12*, 9144–9155. https://doi.org/10.1021/ACSSUSCHEMENG.4C01595.
52. Blumberg, T., Tsatsaronis, G., and Morosuk, T. (2019). On the economics of methanol production from natural gas. Fuel *256*, 115824. https://doi.org/10.1016/J.FUEL.2019.115824.
